# Targeted inhibitors of P-glycoprotein increase chemotherapeutic-induced mortality of multidrug resistant tumor cells

**DOI:** 10.1038/s41598-018-19325-x

**Published:** 2018-01-17

**Authors:** Amila K. Nanayakkara, Courtney A. Follit, Gang Chen, Noelle S. Williams, Pia D. Vogel, John G. Wise

**Affiliations:** 1 The Center for Drug Discovery, Design and Delivery, the Center for Scientific Computing, Southern Methodist University, Dallas, TX 75275-0376 USA; 2 The Department of Biological Sciences, Southern Methodist University, Dallas, TX 75275-0376 USA; 3 Department of Biochemistry, UT Southwestern Medical Center, 5323 Harry Hines Boulevard, Dallas, Texas 75390-9038 United States

## Abstract

Overexpression of ATP-binding cassette (ABC) transporters is often linked to multidrug resistance (MDR) in cancer chemotherapies. P-glycoprotein (P-gp) is one of the best studied drug transporters associated with MDR. There are currently no approved drugs available for clinical use in cancer chemotherapies to reverse MDR by inhibiting P-glycoprotein. Using computational studies, we previously identified several compounds that inhibit P-gp by targeting its nucleotide binding domain and avoiding its drug binding domains. Several of these compounds showed successful MDR reversal when tested on a drug resistant prostate cancer cell line. Using conventional two-dimensional cell culture of MDR ovarian and prostate cancer cells and three dimensional prostate cancer microtumor spheroids, we demonstrated here that co-administration with chemotherapeutics significantly decreased cell viability and survival as well as cell motility. The P-gp inhibitors were not observed to be toxic on their own. The inhibitors increased cellular retention of chemotherapeutics and reporter compounds known to be transport substrates of P-gp. We also showed that these compounds are not transport substrates of P-gp and that two of the three inhibit P-gp, but not the closely related ABC transporter, ABCG2/BCRP. The results presented suggest that these P-gp inhibitors may be promising leads for future drug development.

## Introduction

Despite advances in chemotherapies against cancer, multidrug resistance (MDR) remains a major obstacle to positive therapeutic outcomes in adult^1–3^ as well as pediatric cancers^4^. The most common mechanism of MDR is overexpression of drug efflux transporters of the ATP binding cassette (ABC) family. These pumps reduce the intracellular accumulation of many anticancer drugs to sub-therapeutic levels, thus decreasing or abolishing chemotherapy efficacy. P-glycoprotein (P-gp/ABCB1) is a glycosylated 170-kDa transmembrane protein that is encoded by the MDR1 gene^5^ and is the best studied drug efflux pump of the family of ABC transporters^6^. P-gp is composed of two hydrophobic domains which include 12 transmembrane α-helices that make up the drug binding domains (DBD) and are involved in transporting toxins and xenobiotics out of the cell. Two nucleotide binding domains in the cytoplasmic region are responsible for coupling ATP hydrolysis to the transport processes^7,8^. P-gp is expressed in a variety of normal tissues, such as the intestine, brain, liver, placenta, kidney, and others^9^ and is protective against xenobiotic substances and toxic compounds. It was noted close to 40 years ago that the expression of P-gp is correlated with MDR in many different types of cancers^10^, as well as the lack of response to chemotherapies and poor prognoses in breast^11^ and ovarian^12^ cancers. Overexpression of P-gp in cancers results in reduced accumulation of chemotherapeutics and leads to resistance against many of the currently available anti-cancer drugs such as taxanes (paclitaxel), vinca alkaloids (vinblastine), and anthracyclines (daunorubicin)^13^. The ability of P-gp to transport such diverse chemical classes is at least partly due to multiple transport pathways through the protein which have been recently visualized using molecular dynamics simulations^14^. Studies show that overexpression of P-gp in cancers can be either intrinsic or acquired upon drug treatment, depending on the tissue of origin, for examples see^15–19^. Clinical trials using MDR-inhibitors have had only limited success^20–22^, but the potential of the approach can be appreciated from a trial that used cyclosporine to inhibit P-gp in patients with poor-risk acute myeloid leukemia. Inclusion of the inhibitor with therapy resulted in significant gains in relapse-free and overall survival^23^. The difficulties in clinical trials as discussed in^24,25^ were mainly due to inhibitor toxicities, drug-interactions, and clinical trial design problems. Many of the initial inhibitors were P-gp transport substrates^21,22^, requiring relatively high systemic concentrations for efficacy; others lacked specificity for P-gp and led to drug interactions, for review see^26^. None of these complications, however, diminish the impact or significance that employing effective P-gp inhibitors in cancer chemotherapies would have on patient outcomes.

In earlier work we applied computational searches and detailed three dimensional models of P-gp^27^ to identify small molecules that have the potential to overcome the problems of earlier generation P-gp inhibitors by specifically interacting with the nucleotide binding domains of the pump, while not binding significantly to the drug binding domains^28^. Three compounds were identified (compounds 29, 34 and 45) that caused reversal of paclitaxel resistance in a prostate cancer cell line that over-expresses P-gp^29,30^. Biochemical and biophysical analyses^28^ indicated that compounds 34 and 45 affected nucleotide binding and all three compounds inhibited transport substrate activated ATP hydrolysis by purified P-gp. These results suggested that the inhibitors interacted with the nucleotide binding domains and not the drug binding domains and had the potential of not being transport substrates for P-gp. In the present study we extended our investigation of the reversal of multidrug resistance by these compounds to cancers of different origins using both 2-dimensional cell culture and spheroid – microtumor assays. We demonstrated that co-administration of these agents with chemotherapeutics resulted in significantly increased microtumor penetration of the fluorescent P-glycoprotein transport substrate, calcein AM, as well as increased accumulation of calcein AM or daunorubicin in two-dimensional cell culture studies. The studies show that the inhibitors directly blocked the pumping action of P-glycoprotein, but were not pump substrates themselves. Two of the three compounds are P-gp specific, while the third also inhibited to a lesser degree a second ABC transporter, the breast cancer resistance protein (BCRP, ABCG2). Cell mortality in both 2D and spheroid cultures was markedly increased when chemotherapeutics were used in combination with any of these P-glycoprotein inhibitors. Cell migration was also strongly inhibited. Protein expression analyses showed that the compounds did not downregulate P-gp expression under the conditions used for re-sensitizing the MDR cancer cells. These properties of the P-gp inhibitors studied here make them attractive leads for further development.

## Results

### Overexpression of P-glycoprotein leads to multidrug resistance in the ovarian cancer cell line, A2780ADR

Follit *et al*.^29^ showed that the addition of compounds 29, 34, or 45 previously identified in^28^ to a prostate cancer cell line that over-expresses P-gp^30^ caused reversal of the MDR phenotype. In the present study, we expanded our work to a drug resistant ovarian cancer cell line to assess whether the observed effects were cancer cell type specific or whether they might be more generally applicable. Fig. S1 (supplemental information) shows the characterization of the paired ovarian cancer cell lines, A2780^31^, and the highly drug resistant line derived from it, A2780ADR^32^. Western blot analyses using a P-gp-specific primary antibody showed that while the A2780ADR cells expressed significant amounts of P-gp (Fig. S1A, left lane), no P-gp was detectable in the parental A2780 cells (supplemental Fig. S1A, right lane). Original Western blots are shown in Fig. S2 as required by the journal. Also consistent with earlier work^32^, A2780ADR cells showed much higher resistance than the parental A2780 cell line to the vinca alkaloid, vinblastine, when tested using a resazurin cell viability assay^33,34^ (Fig. S1B). High levels of resistance to paclitaxel by A2780ADR cells were also observed when exposing the cells to increasing concentrations of the taxane, paclitaxel (Fig. S1C). Inclusion of 60 µM novobiocin, a relatively specific inhibitor of BCRP^35^, or 250 µM probenecid, an inhibitor of the multidrug resistance associated protein 1 (ABCC1, MRP-1)^36^, together with vinblastine had no effect on the sensitivity of A2780ADR cells to the chemotherapeutic (Fig. S1B). These results strongly suggest that the A2780ADR ovarian cancer cell line was phenotypically multidrug resistant and that this MDR phenotype was correlated to overexpression of P-glycoprotein and not BCRP or MRP-1.

### Inhibitors of P-glycoprotein reverse MDR phenotype of the ovarian cancer cell-line, A2780ADR

**Figure 1:**
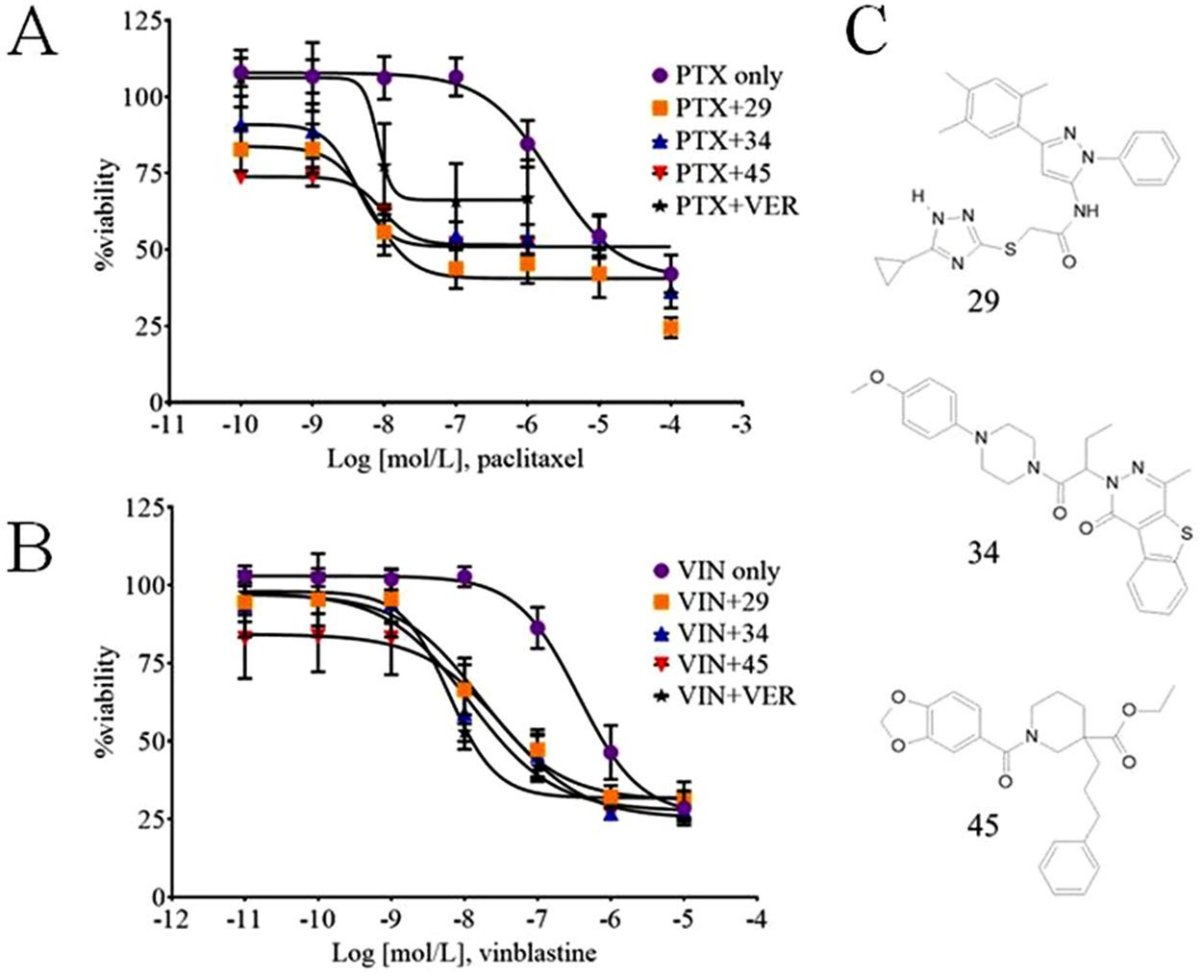
Reversal of paclitaxel or vinblastine resistances by novel inhibitors of P-glycoprotein using metabolic viability assays. A2780ADR cells were treated in the presence of compounds 29, 34, 45 or verapamil with the indicated concentrations of chemotherapeutic. Panel A: circles, paclitaxel alone; squares, paclitaxel plus 25 µM compound 29; triangles, paclitaxel plus 25 µM compound 34; inverted triangles, paclitaxel plus 25 µM compound 45; stars, paclitaxel plus 25 µM P-gp substrate verapamil. Panel B: circles, vinblastine alone; squares, vinblastine plus 10 µM compound 29; triangles, vinblastine plus 10 µM compound 34; inverted triangles, vinblastine plus 10 µM compound 45; stars, vinblastine plus 10 µM P-gp substrate verapamil. Data represents the mean ± SD of 12 replicates from two independent experiments. Panel C: Chemical structures of novel P-gp inhibitors 29, 34 and 45. PTX, paclitaxel; VIN, vinblastine; VER, verapamil.

Figure 1 shows the relative viability of A2780ADR cells as reported by the resazurin assay when incubated with increasing concentrations of paclitaxel (Fig. 1A) or vinblastine (Fig. 1B) with or without addition of P-gp inhibitors 29, 34, 45, or verapamil. The structures of the compounds 29, 34 and 45 are shown in Fig. 1C. It can be seen from Fig. 1A,B that the sensitivity of the MDR cell line to chemotherapeutics was increased by several orders of magnitude in the presence of the P-gp inhibitors. Inclusion of the BCRP inhibitor, novobiocin, or the MRP-1-inhibitor, probenecid, had no discernable effect on these cells (Table 1), suggesting that neither BCRP nor MRP-1 contributed to the MDR phenotype of the cells. Table 1 also compares the calculated IC50 values for the chemotherapeutics paclitaxel or vinblastine in the presence or absence of P-gp inhibitors for the two ovarian cancer cell lines. No sensitization of the non-MDR parental cell line A2780 was observed. These results suggest that the ovarian cell line A2780ADR is multidrug resistant because of overexpression of P-gp and that its MDR phenotype can be reversed by inhibitors of P-gp ATPase activity, compounds 29, 34 and 45^28,29^, as well as the P-gp transport substrate and competitive transport inhibitor, verapamil.

**Table 1:** *In silico* identified P-gp inhibitors reverse MDR phenotype of ovarian cancer cell line.

		paclitaxel IC_50_ (nmol/L)	Fold sensitization	vinblastine IC_50_ (nmol/L)	Fold sensitization
A2780ADR	paclitaxel/vinblastine only	2146		361	
	+ compound 29				
	5 μM	121	18	52	7
	10 μM	42	52	16	22
	25 μM	7	308		
	+ compound 34				
	5 μM	57	38	42	9
	10 μM	20	107	12	31
	25 μM	4	528		
	+ compound 45				
	5 μM	122	18	49	7
	10 μM	90	24	34	11
	25 μM	10	225		
	+ verapamil				
	5 μM	53	41	19	19
	10 μM	22	99	6	58
	25 μM	8	265		
	+ novobiocin				
	60 μM			475	1
	+ probenecid				
	250 μM			525	1
A2780	paclitaxel/vinblastine only	5		15	
	+ compound 29				
	10 μM	5	1	14	1
	+ compound 34				
	10 μM	5	1	14	1
	+ compound 45				
	10 μM	4	1	16	1
	+ verapamil				
	10 μM	5	1	13	1

IC_50_ concentrations of chemotherapeutics which resulted in 50% decrease in A2780ADR viability were calculated from data as shown in Fig. 1, S3 and S4 using a nonlinear, four-parameter curve fitting procedure.

### Novel P-gp inhibitors increased apoptosis in MDR prostate cancer cells when co-treated with paclitaxel

**Figure 2:**
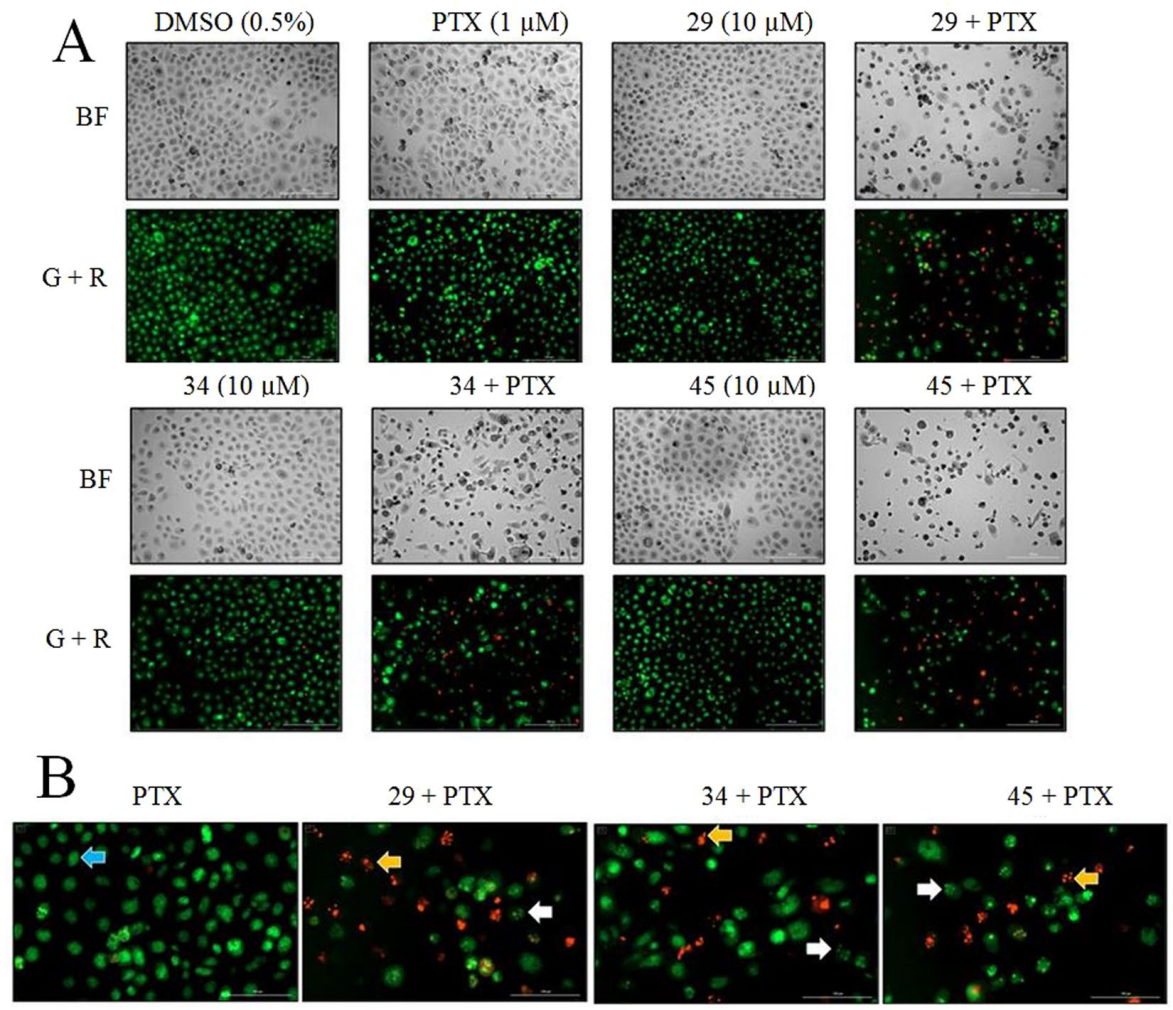
Reversal of paclitaxel resistance by novel P-gp inhibitors induces apoptosis in MDR prostate cancer cells. Panel A: DU145TXR cells were treated either with 1 μM paclitaxel alone, 10 μM of novel P-gp inhibitors alone, or combinations of paclitaxel with P-gp inhibitors for 48 hours, followed by staining with acridine orange and ethidium bromide. Top panels show the bright field images while lower panels show the merged images obtained using GFP and Texas Red fluorescence filters. Both were recorded with 10X objectives. Panel B. Enlarged images of the merged images obtained in panel A with the treatments shown. The *blue arrow* represents a nucleus that is not affected by paclitaxel. Cells in early stages of apoptosis with visible nuclear fragmentation are indicated with *white arrows*, and apoptotic cells, stained red with ethidium bromide are indicated by *yellow arrows*. PTX, paclitaxel; BF, bright field photomicrograph; G + R, green plus red fluorescence composite images.

The results of the resazurin viability assays with the A2780ADR cells (Fig. 1 and S1) showed unexpectedly high residual cell viabilities of between 25% with vinblastine and up to 60% when paclitaxel was used. While the overall results were consistent with increased cytotoxicity of chemotherapeutics in the presence of P-gp inhibitors, these results did not directly demonstrate that co-administration with inhibitor led to increased cell mortality. To test for increased cell mortality, the adherent MDR prostate cancer cells (DU145TXR^30^) were used, since the semi-adherent properties of A2780ADR cells made cell imaging much less reliable. In experiments designed to assess the induction of apoptosis, DU145TXR cells were treated either with 1 μM paclitaxel alone, 10 μM P-gp inhibitors alone, or combinations of paclitaxel with inhibitors for 48 hours. Analysis of these assays took advantage of the facts that acridine orange is taken up both by viable and non-viable cells, intercalates with double stranded DNA, and emits green fluorescence, while ethidium bromide is only taken up by non-viable cells and emits a strong red fluorescence after interchelating with DNA^37,38^. As shown in Figure 2A, cells treated with vehicle, paclitaxel or P-gp inhibitors alone showed green fluorescence with highly organized nuclear morphologies, suggesting no induced apoptosis. In Fig. 2B, the blue arrow points out one such morphologically non-apoptotic nucleus. Upon combination treatment with chemotherapeutic and P-gp inhibitors, the number of cells with shrunken, rounded, and darker condensed cell morphologies increased (Fig. 2A, bright field panels). The number of cells that demonstrated obvious chromatin fragmentation also increased (Fig. 2B, white arrows). The number of dead cells, as indicated by ethidium bromide fluorescence also increased after co-treatments with chemotherapeutic and P-gp inhibitors (Fig. 2B, yellow arrows). These results indicated that paclitaxel induced apoptosis in DU145TXR cancer cells when P-gp activity was blocked by the P-gp targeted inhibitors.

### Reversal of MDR by added P-gp inhibitors causes inhibition of cell proliferation upon exposure to previously sub-lethal concentrations of chemotherapeutics

**Figure 3:**
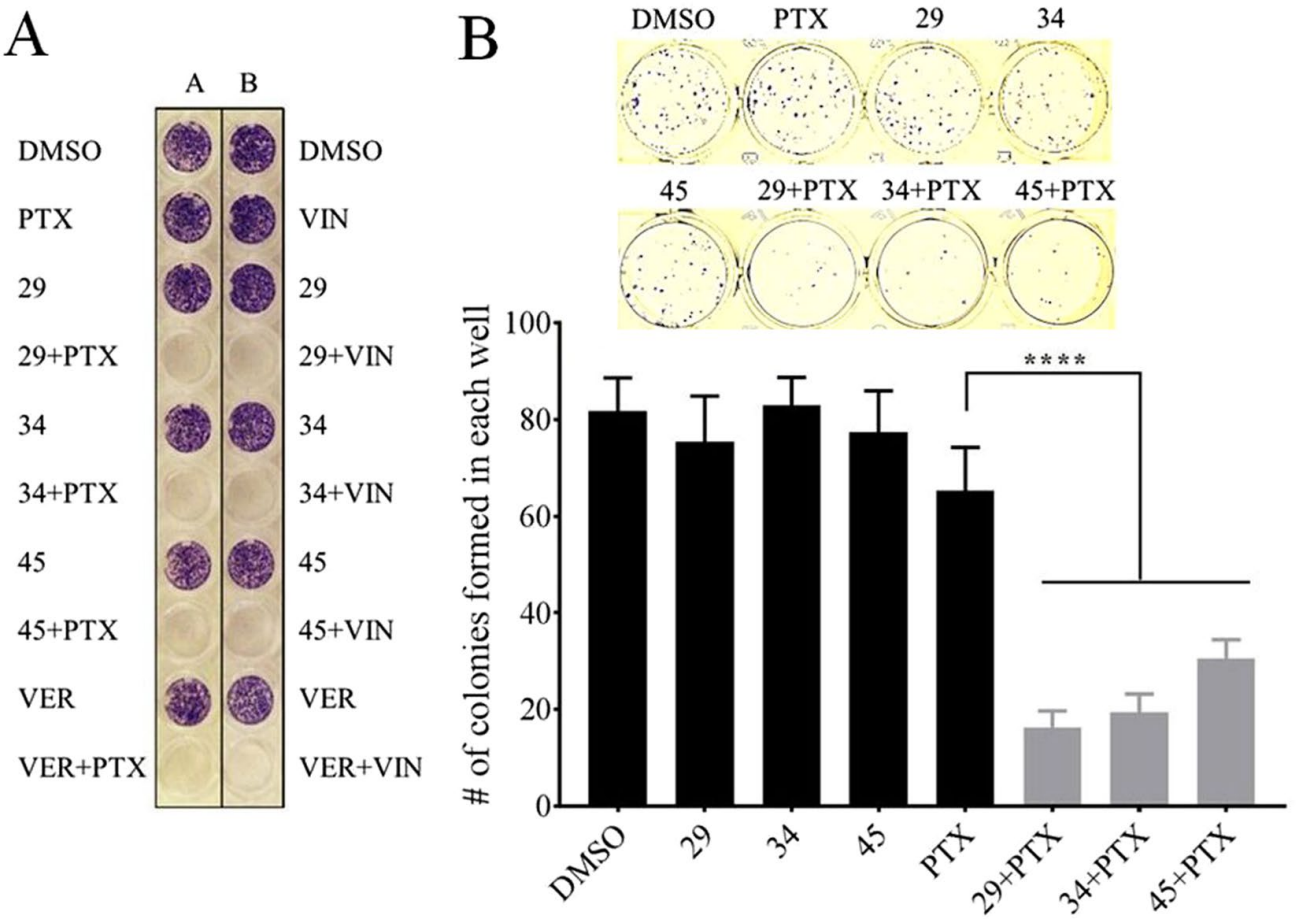
Reversal of chemotherapy resistances by novel inhibitors of P-glycoprotein. Panel A: Qualitative colony formation analyses using multidrug resistant ovarian cancer cells. A2780ADR cells were treated with chemotherapeutics and/or inhibitors as indicated in the figure for 48 hrs, washed and subsequently cultured for an additional 96 hours. Remaining cell colonies were stained with crystal violet. *Left column from top to bottom:* vehicle (DMSO); vinblastine (VIN) alone; compound 29 alone; vinblastine and compound 29; compound 34 alone; vinblastine and compound 34; compound 45 alone; vinblastine and compound 45; verapamil (VER) alone; vinblastine and verapamil. *Right column from top to bottom:* vehicle (DMSO); paclitaxel (PTX) alone; compound 29 alone; paclitaxel and compound 29; compound 34 alone; paclitaxel and compound 34; compound 45 only; paclitaxel and compound 45; verapamil (VER) alone; paclitaxel and verapamil. The concentrations used were 0.1 µM vinblastine, 1 µM paclitaxel, 10 µM of inhibitors 29, 34, 45 or verapamil. Panel B: Quantitative colony formation analyses using multidrug resistant prostate cancer cells. The experiments were performed as above, except that DU145TXR cells were seeded and grown to lower densities than in (**A**) and exposure to chemotherapeutic and inhibitors was for 24 hours at lower inhibitor concentrations. *Top:* Images of a representative experiment showing stained colonies after 5 days of recovery. Treatments were performed with 5 µM of P-gp inhibitors 29, 34 or 45 alone, 0.5 µM paclitaxel (PTX) alone, or in combination. *Bottom*: Quantitative analysis of colonies formed in (**B**) Each histogram represents the average ± S.D. (n = 6, three replicates from two individual experiments; ****P < 0.0001).

To experimentally test the hypothesis that cells which had residual metabolic activity after co-treatment with chemotherapeutic and P-gp inhibitors were either dying or lost proliferation ability, colony formation experiments^39^ were performed. Figure 3A shows the results of qualitative colony formation experiments, where A2780ADR cells were treated for 48 hours with either vehicle, 0.1 µM vinblastine, 1 µM paclitaxel or 10 µM P-gp inhibitor alone, or combinations of chemotherapeutic and P-gp inhibitor. Time of exposure to chemotherapeutic and / or inhibitor were the same as in Fig. 1. The cells were then washed with media that did not contain chemotherapeutic or inhibitors and were allowed to recover for 96 hours after which the presence of cell colonies was assessed by crystal violet staining. In the presence of inhibitor in combination with paclitaxel or vinblastine, no cell colonies were observed (Fig. 3A). In contrast, exposure to paclitaxel, vinblastine, or the inhibitors alone, resulted in very dense, viable cell colonies which were qualitatively equivalent to the DMSO control (Fig. 3A).

To more quantitatively assess cell viability and colony formation and to test a different multidrug resistant cancer cell line, similar experiments were performed using the MDR prostate cancer cell line, DU145TXR^30^. The conditions for these experiments were chosen to represent the lowest exposure time and inhibitor concentration that resulted in significant differences in the number of colonies formed. Cells were treated for 24 hours with 0.5 µM paclitaxel alone, 5 µM inhibitor alone, or combinations of inhibitor and chemotherapeutic. Afterwards, the media containing inhibitor and/or chemotherapeutic were removed and the cells were allowed to recover for 120 h in the absence of chemotherapeutic or P-gp inhibitor. The cells were fixed and stained as described above. Figure 3B, top, shows images of the crystal violet stained colonies visible to the unaided eye. Figure 3B bottom shows the statistical analyses of two independent experiments. The number of colonies formed in the presence of paclitaxel and P-gp inhibitors was found to be significantly lower than when cells were grown with inhibitor or chemotherapeutic alone. These results support the hypothesis that the residual metabolic activities reported by the resazurin viability assays in Fig. 1 and S1 were due to the residual metabolic activities of cells that were dying, but not yet dead.

### P-glycoprotein inhibitors prevent multidrug resistant cancer cells from migrating when exposed to chemotherapeutics that interrupt microtubule dynamics

**Figure 4:**
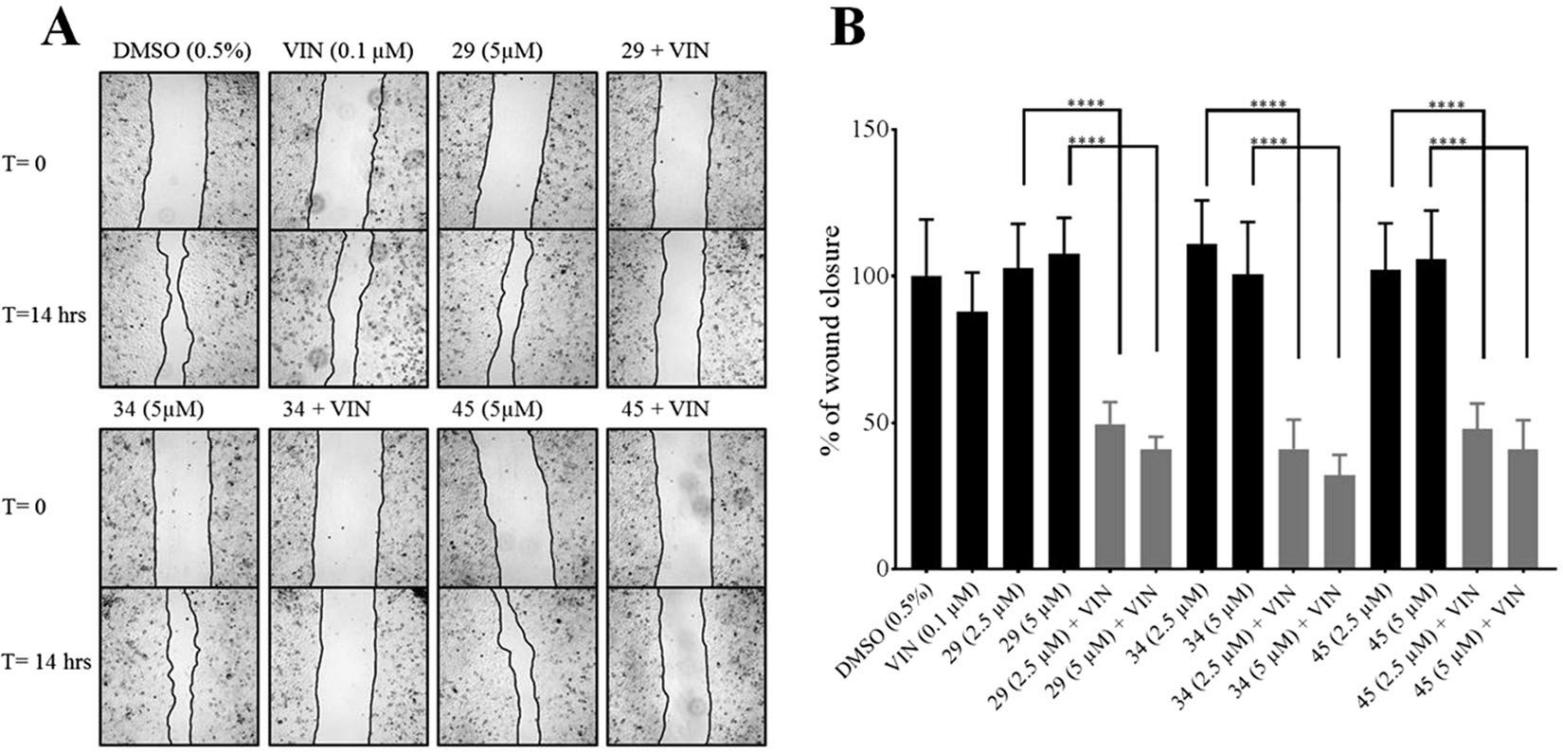
P-gp inhibitors prevent the migration of MDR cancer cells in the presence of chemotherapeutics that target microtubule dynamics. Panel A: Wound healing assays. Confluent monolayers of the MDR prostate cancer cell line, DU145TXR, were manually scratched and subsequently cultured for 14 hours under conditions that inhibited cell proliferation. Representative 4X bright field micrographs of the scratch zones were recorded. Closure of the scratches was then evaluated in the presence of chemotherapeutic vinblastine (VIN) alone, P-gp inhibitors 29, 34 or 45 alone, as well as in the indicated combinations. The edge of the wound is marked by a black line. Panel B: Percentage wound closure. The average percentage of wound closure under different treatment conditions was compared to vehicle treated. Data are expressed as average ± S.D. of duplicate experiments (n = 12; ****P < 0.0001). DMSO, carrier vehicle only; VIN, 0.1 µM vinblastine; 29, 34, or 45 indicates added P-gp inhibitor at 2.5 or 5 µM.

To assess whether the P-gp inhibitors affect cancer cell migration in the presence of chemotherapeutics, wound healing assays^40–42^ were performed with the MDR prostate cancer cell line^30^. Figure 4 shows the results of these wound healing assays under conditions of limited cell proliferation in the absence or presence of 0.1 µM vinblastine and 5 µM inhibitor 29, 34, or 45. Controls with the P-gp inhibitors alone (no chemotherapeutic present) are also shown. Figure 4A shows micrographs typical of the scratch zones immediately after the injury (zero time) and after 14 hours of incubation in low serum media. Figure 4B presents the averages of the relative wound closures normalized to wound closure in the presence of vehicle only (DMSO). Addition of vinblastine or the P-gp inhibitors by themselves resulted in reduction of the area of the scratch wound similar to vehicle controls, indicating that the MDR cancer cells were able to migrate into the wound site and close the scratch gap under these non-proliferative conditions. When any of the three P-gp inhibitors were used in combination with vinblastine, significant inhibition of wound healing was observed, suggesting that cancer cell migration was strongly inhibited. The closing of scratch area was limited to between 41% (vinblastine with 29 or 45) and 32% (vinblastine with 34) of those of the vehicle only controls. There was no significant difference when 2.5 or 5 µM of inhibitor was used in the presence of chemotherapeutic.

### The intracellular retention of transport substrates of P-glycoprotein is enhanced in the presence of P-gp inhibitors in MDR cancer cells over-expressing P-gp

The results presented in Figs 1–4 suggested that P-glycoprotein inhibition leads to enhanced therapeutic efficacies. To determine whether the P-gp inhibitors caused increased cellular retention of P-gp transport substrates, we assessed the accumulation of a known P-gp substrate, calcein AM. Calcein AM is an uncharged, acetoxymethyl derivative of the anionic fluorescent dye calcein and is known to be a substrate for P-gp^43^. Cellular esterases convert calcein AM to calcein, which is not transported by P-glycoprotein, making it a useful fluorescent probe for P-gp transport activity.

**Figure 5:**
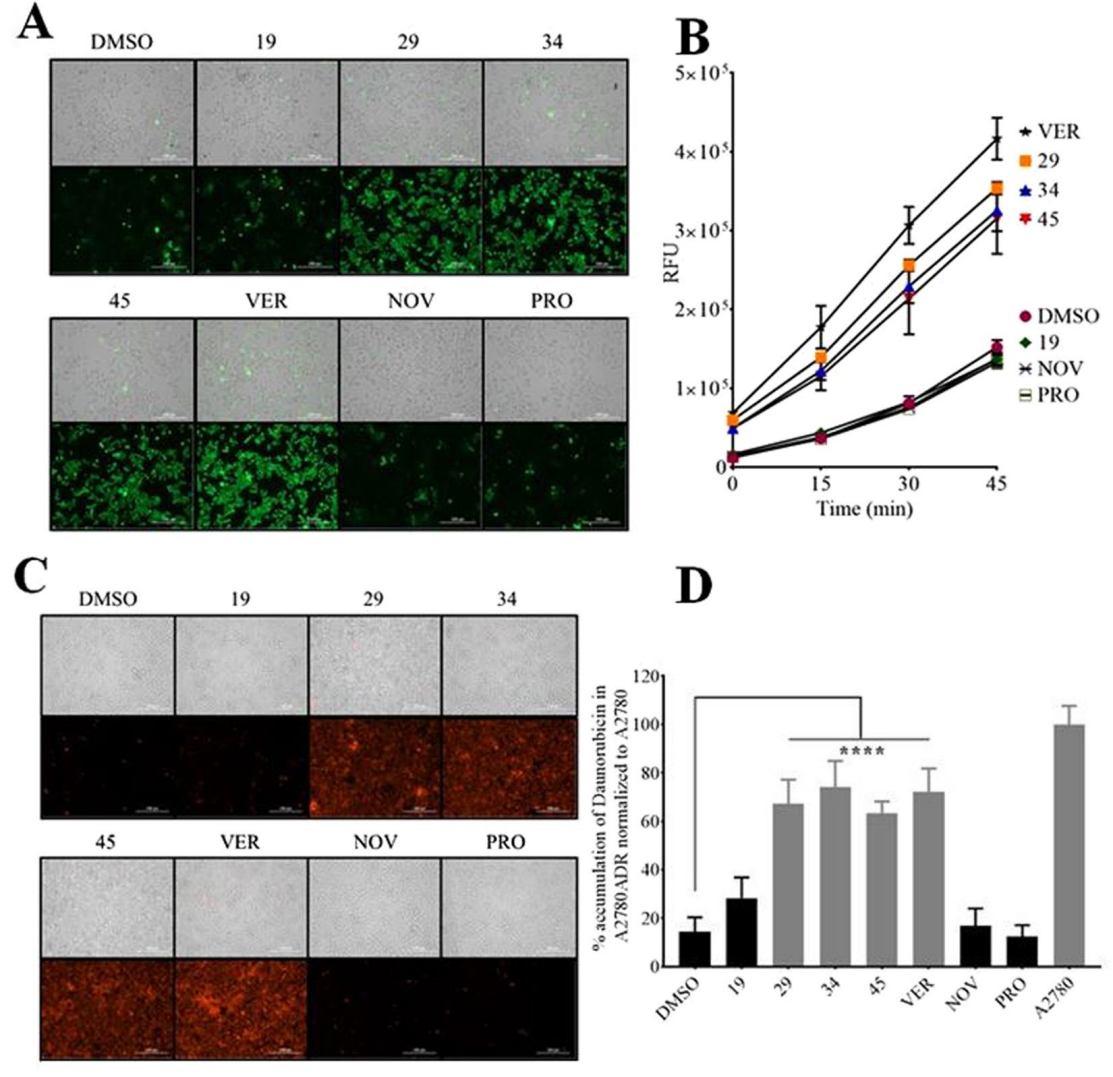
P-gp inhibitors restore calcein-AM or daunorubicin accumulation in MDR ovarian cancer cells. Panel A: Calcein-AM accumulation. Calcein AM accumulation upon P-gp inhibition was analyzed as described in methods using A2780ADR cells. All experiments had identical components except for the additions indicated. Additions were: DMSO, carrier vehicle at 0.5% final volume; compounds 19, 29, 34 or 45 at 10 µM; VER, 10 μM verapamil; NOV, 60 μM novobiocin; PRO, 250 μM probenecid. Scale bars are 200 µm. Panel B: Time course of calcein accumulation. Fluorescence measurements were made on the entire wells during the imaging experiments described in panel A. The increase in relative fluorescence resulting from accumulated calcein is plotted versus the time of incubation. The indicated additions were as described in panel A. Panel C: Daunorubicin accumulation. Intracellular daunorubicin accumulation in A2780ADR cells was observed similarly to the accumulation of calcein (see panel A). After a 2 hour incubation with 10 µM daunorubicin, fluorescence images of the cells were obtained using a Texas Red filter. Additions were as indicated in panel A. Panel D: Quantification of intracellular daunorubicin accumulation. A2780ADR cells were incubated as described for panel C. After the 2 hour incubation, cells were washed twice with cold RPMI to remove extracellular daunorubicin, and then lysed. The fluorescence of each well was measured at excitation 488/20 nm and emission 575/20 nm. Percent accumulation of daunorubicin in A2780ADR cells was normalized to the parental A2780 cells. Additions were as indicated in panel A. Each histogram represents the average ± S.D. from two independent experiments (n = 6; ****P < 0.0001).

Figure 5A shows that inclusion of P-gp inhibitors 29, 34, or 45 in media containing calcein AM with the P-gp overexpressing ovarian cancer cell line, A2780ADR, resulted in significant increases in intracellular calcein as detected by its green fluorescence. Compound 19 had been identified in earlier studies as an inhibitor of P-gp ATPase activity in cell-free biochemical assays^28^, but was shown to be ineffective in reversing multidrug resistance in cell-based assays^29^. This is likely due to a negative charge of the molecule at neutral pH, making it unable to enter intact cells. Inclusion of compound 19 to the experiments described here served as a negative control. Addition of the P-gp transport substrate verapamil also led to significant increases in intracellular fluorescence, suggesting that uninhibited P-glycoprotein activity in these cells was responsible for the low intracellular calcein accumulation observed in the absence of added P-gp inhibitors or substrates. Addition of the BCRP inhibitor, novobiocin, or the MRP-1 inhibitor, probenecid, did not lead to increased intracellular fluorescence, suggesting that BCRP and MRP-1 were not responsible for removing calcein AM from these cells. Time courses for the changes in calcein fluorescence intensities in the presence of the different inhibitors are shown in Figure 5B. The data indicate that both increased rates of accumulation as well as increased overall intracellular concentrations of calcein were achieved when P-gp specific inhibitors were present.

It is worth noting that a number of cells in the uninhibited controls (Figure 5A and B) also showed a few isolated puncta of fluorescence. This is hypothesized to be a consequence of subpopulations of cells in the MDR cultures that are not over-expressing P-gp. Given the plasticity of cancer cell genetics, this observation may be expected. Inclusion of chemotherapeutics would be expected to decrease the number of these calcein positive cells in the uninhibited control experiments.

The differential accumulation of the chemotherapeutic daunorubicin in the MDR ovarian cancer cells in the presence and absence of various multidrug resistance pump inhibitors was assessed next. Similar in design to the calcein AM accumulation experiments described, these experiments took advantage of the intrinsic red fluorescence of daunorubicin. Figure Fig. 5C shows significant increases in intracellular daunorubicin fluorescence when the cells were treated with daunorubicin in combination with 10 µM inhibitor 29, 34, or 45. Inclusion of BCRP- or MRP-1 specific inhibitors (60 µM and 250 µM, respectively) did not result in observable increases in intracellular daunorubicin fluorescence suggesting that these effects were dependent on the specific inhibition of P-glycoprotein. Figure 5D presents the quantification of daunorubicin accumulation as assayed in Fig. 5C. The accumulation of daunorubicin in the presence of the experimental P-gp inhibitors was comparable to that observed for the non-MDR parental cell line, A2780 (Fig. 5D).

### Increased accumulation and deep penetration of calcein in 3D tumor spheroids treated with P-gp inhibitors

**Figure 6:**
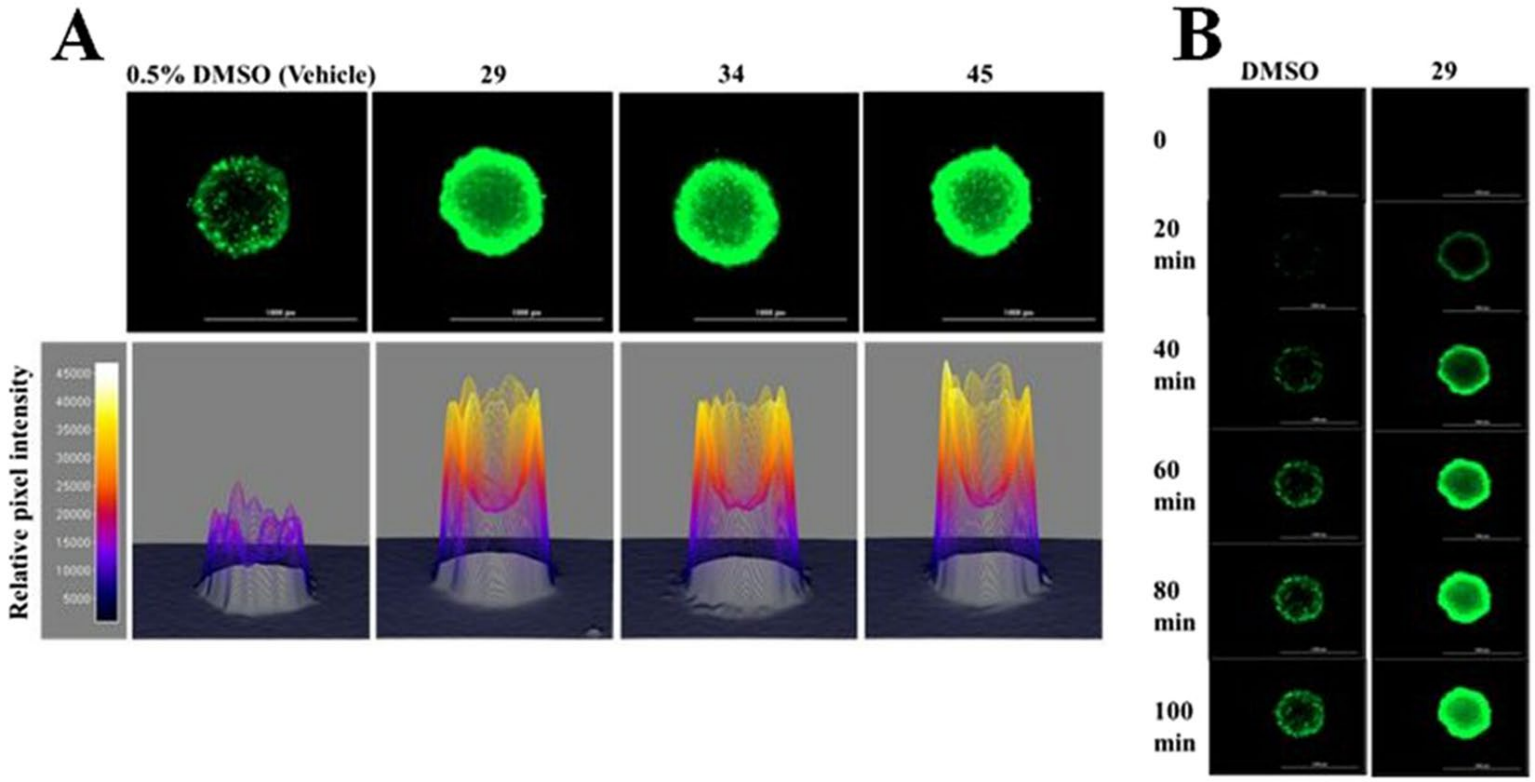
P-gp inhibitors increase the accumulation and penetration of calcein-AM in MDR prostate cancer spheroids. Panel A: Calcein accumulation in 3D-spheroids. Upper panel – After 100 minutes of incubation with 2.5 µM of the P-gp substrate calcein-AM, fluorescence images of the spheroids were recorded. DMSO, 0.5% final volume; compounds 29, 34 or 45 at 15 µM. Scale bar indicates 1000 µm. Lower panel – 3D surface plots representing the pixel intensities of the corresponding images from the experiments above. Panel B: Time course of calcein accumulation. Fluorescence images of the spheroids treated with vehicle only or P-gp inhibitor 29 were obtained over 20 minute intervals using a GFP filter as described in panel A. Increases in calcein accumulation in the presence of compound 34 or 45 were observed to be similar to those shown with compound 29 (data not shown).

To assess the ability of the targeted P-gp inhibitors to facilitate the penetration of P-gp substrates into cells in tumor-like structures, microtumor spheroid cultures of an MDR prostate cancer cell line^30^ were produced. Incubation of the spheroids with calcein AM in the presence of either vehicle alone or P-gp inhibitor 29, 34, or 45 showed considerably increased calcein fluorescence (Fig. 6A top row). The relative calcein fluorescence was visualized as 3D surface plots using the pixel intensities of the corresponding images (Fig. 6A, lower panel). Calcein accumulation was higher in the interior regions of the microtumors in the presence of P-gp inhibitors than in the DMSO control. These experiments suggest that calcein penetrated deeper into the interior of the microtumor in the presence of P-gp inhibitors. The time dependence of calcein AM uptake and calcein accumulation in the presence or absence of compound 29 is shown in Fig. 6B.

### Increased cytotoxicity of daunorubicin in the presence of P-glycoprotein inhibitors in multidrug resistant prostate cancer microtumors

**Figure 7:**
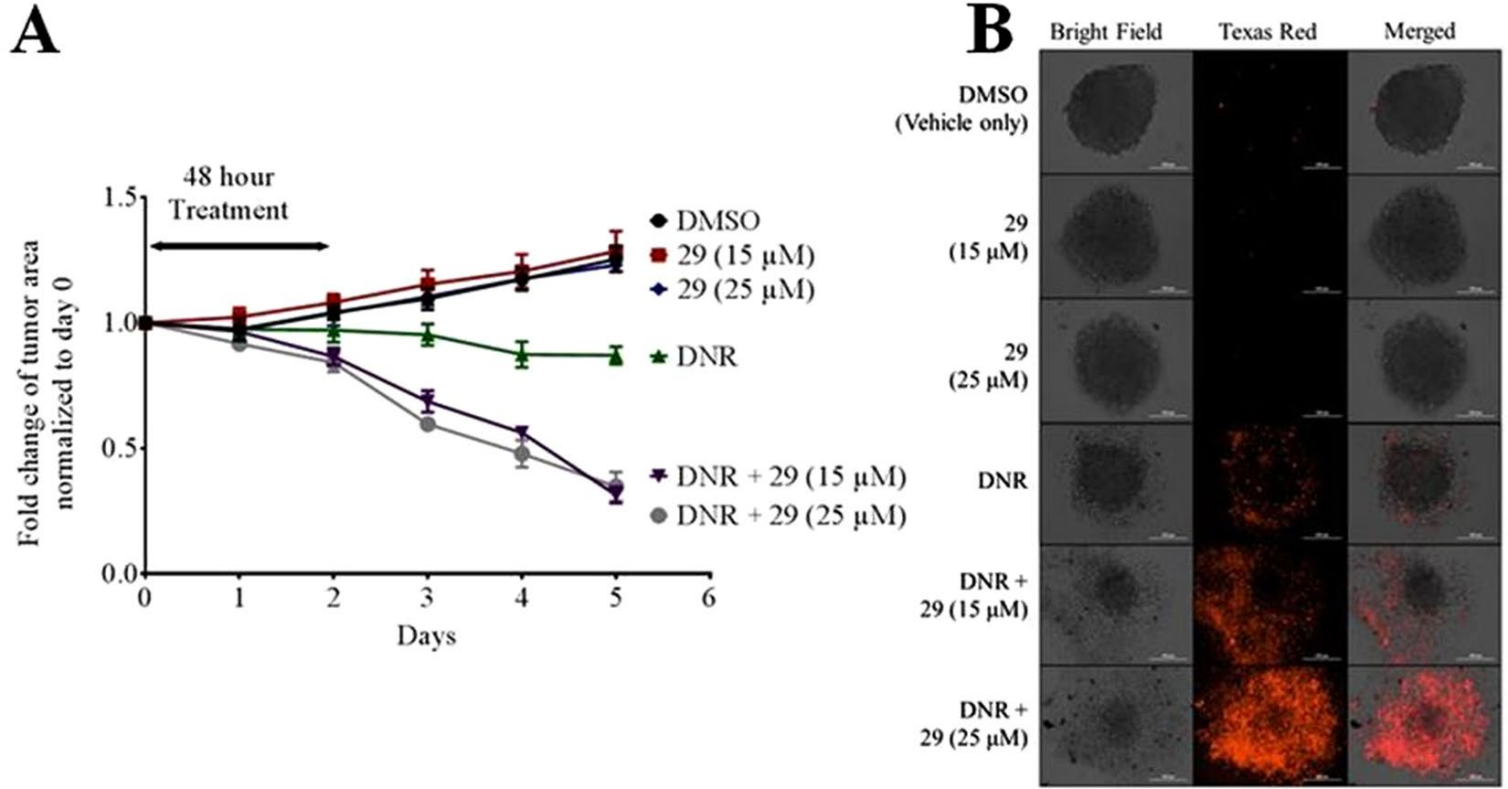
Inhibition of P-gp leads to increased daunorubicin-induced cell death in MDR spheroid microtumors. Panel A: Time course of changes in tumor area. MDR DU145TXR spheroids were prepared and treated with P-gp inhibitory compound 29 with or without daunorubicin as described in methods. Areas of the spheroids were calculated at each day of the experiment and fold change plotted versus time. Values were normalized to the size of the tumor before addition of chemotherapeutic. Each point represents average ± S.D. (n = 4). Panel B: Photomicrographs of spheroids at the end of the experiment. At the end of the experiment, dead cells were illuminated by ethidium bromide staining and bright field and fluorescence (Texas Red channel) micrographs of typical spheroids were recorded. Treatments were as indicated in the panel labels. For each spheroid shown, bright field (left), Texas Red fluorescence (middle) and merged (right) images are shown. DNR, daunorubicin.

The experiments described in Figs 1–3, S1, S3 and S4 showed that the cytotoxicity of chemotherapeutics like paclitaxel or vinblastine to MDR cancer cells in traditional 2D cell culture was increased in the presence of the P-gp inhibitors 29, 34, or 45. It was of interest to test whether combination treatment of P-glycoprotein inhibitors with chemotherapeutic would increase the cytotoxicity of chemotherapeutics in microtumors. To investigate this hypothesis, DU145TXR spheroids were treated for 48 hours with either vehicle, P-gp inhibitor 29 or daunorubicin, as well as the combination of daunorubicin with two different concentrations of compound 29. After treatment, reagents were removed and the spheroids were incubated with fresh complete media for an additional 4 days. The growth of the spheroids under these conditions is shown in Fig. 7A. The change in size of the microtumors was quantified as the surface area of the spheroids as observed in the bright field images. Growth of the tumors in the presence of 15 or 25 µM of 29 alone was similar to that in the presence of DMSO alone, demonstrating the low toxicity of 29. In the presence of 1 µM daunorubicin, the tumor size did not change significantly over the course of the experiment, while combination with 15 or 25 µM of 29 led to a ~ 60% reduction in the size of the microtumors. Figure 7B shows a representative set of photomicrographs that correspond to the end-points of these experiments. The left panel shows bright field images of the microtumors after the treatments indicated in the figure. The middle panel shows the red fluorescence of the same microtumors upon addition of ethidium bromide, which stains dead cells^37^. Merged images are shown in the right panel. The results indicate that the efficacy of cancer cell killing by the daunorubicin chemotherapy treatment of these microtumors was increased in the presence of the P-glycoprotein inhibitor 29.

### P-gp inhibitors 29, 34 and 45 do not affect P-gp protein expression levels

Previous studies have shown that reversal of multidrug resistances in cancers can sometimes be due to lowered expression of the protein and not to direct inhibition of P-gp transport by an experimental compound^44,45^. To test whether the inhibitors used in this study affected P-gp expression, Western blot analyses of the P-gp overexpressing prostate cancer cell line were performed after incubation for 48 hours with inhibitors 29, 34, or 45. These conditions led to at least 17-fold sensitization of the DU145 TXR cells to paclitaxel^29^. The results of the Western blot analyses are shown in Figs S5 and S6. No decreases in P-gp protein expression were observed.

### P-gp inhibitors 29, 34 and 45 are not transport substrates of P-gp

**Figure 8:**
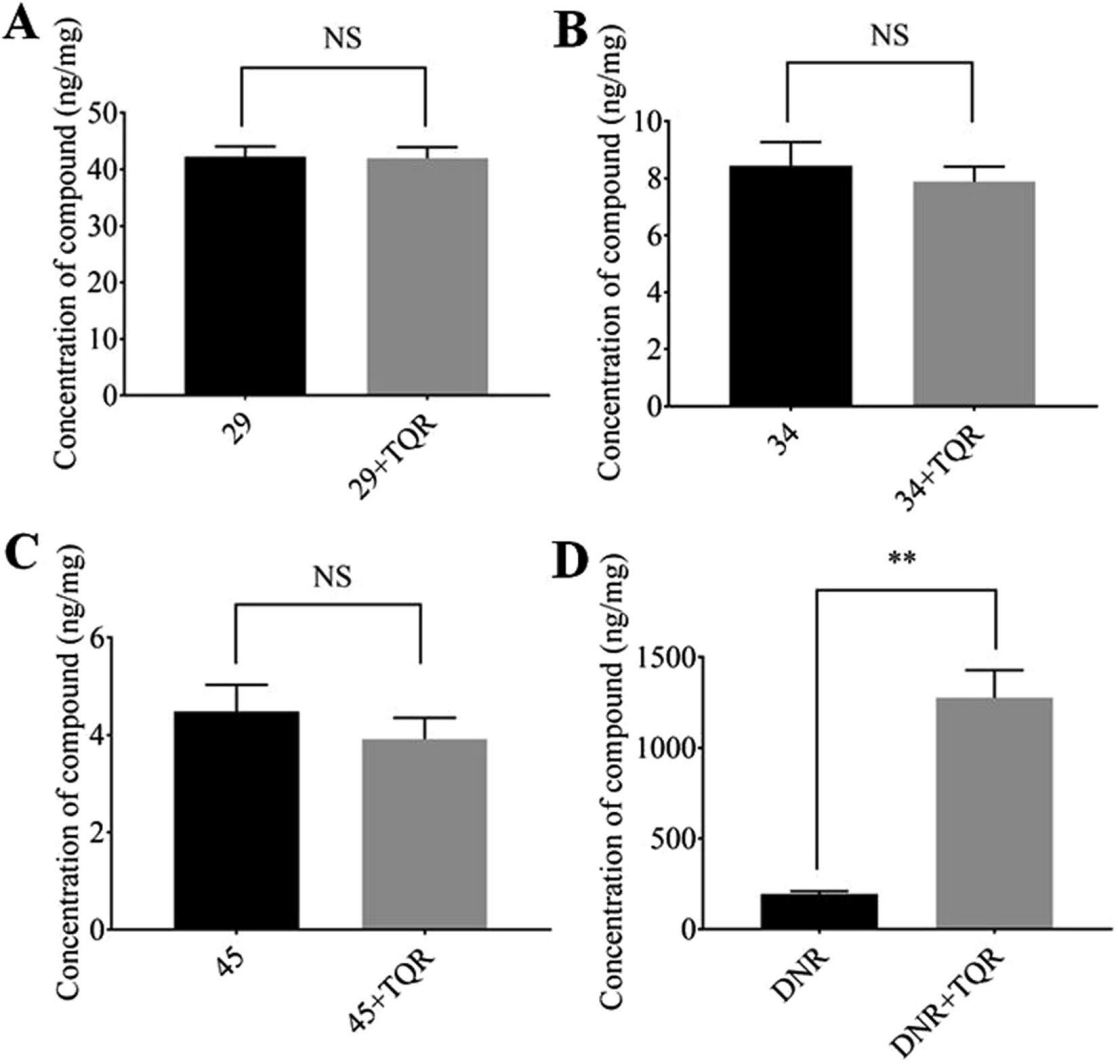
Compounds 29, 34 and 45 are inhibitors of P-gp and not transport substrates. Quantitative LC-MS/MS analysis of intracellular accumulation of 29 (panel A), 34 (panel B), 45 (panel C), or daunorubicin (panel D) in DU145 TXR. Each histogram represents the average ± S.D. (n = 3, two independent experiments); **P < 0.01; NS – not significant). DNR, daunorubicin; TQR, tariquidar.

The original premise of our search for P-gp inhibitors was that compounds that are not transport substrates of the pump would make better lead compounds for future development for clinical use^28,29^. To test whether compounds 29, 34 and 45 are transport substrates, accumulation assays were performed where DU145TXR cells were incubated with 5 µM of 29, 34 and 45 in the presence or absence of the known P-gp inhibitor tariquidar^46,47^. A P-gp transport substrate, daunorubicin, was used as a positive control. After incubation for 2.5 hours, cells were washed with ice-cold buffer and counted. After cell lysis, the cell contents were analyzed by LC-MS/MS. The results of these analyses are shown in Fig. 8. The studies indicate that cellular accumulation of compounds 29, 34 and 45 was no different in the presence or absence of tariquidar, while cellular accumulation of the P-gp substrate, daunorubicin, was significantly increased in the presence of tariquidar. These results support the hypothesis that compounds 29, 34 and 45 are not P-gp transport substrates.

### Inhibitors 34 and 45 are P-gp specific, while compound 29 also affects the breast cancer resistance protein

In order to assess whether the inhibitors were specific for P-gp or would also inhibit other ABC transporters, we created a BCRP-overexpressing breast cancer cell line, MCF-7 M100, which we derived from MCF-7 cells^48^ by exposing the cells to increasing, sub-lethal concentrations of the BCRP pump substrate and chemotherapeutic, mitoxantrone^49^. Fig. S7 shows the results of Western blot analyses of cell lysates of the MCF-7 and MCF-7 M100 cell lines indicating that the MCF-7 M100 derivative line overexpresses the BCRP protein.

**Figure 9:**
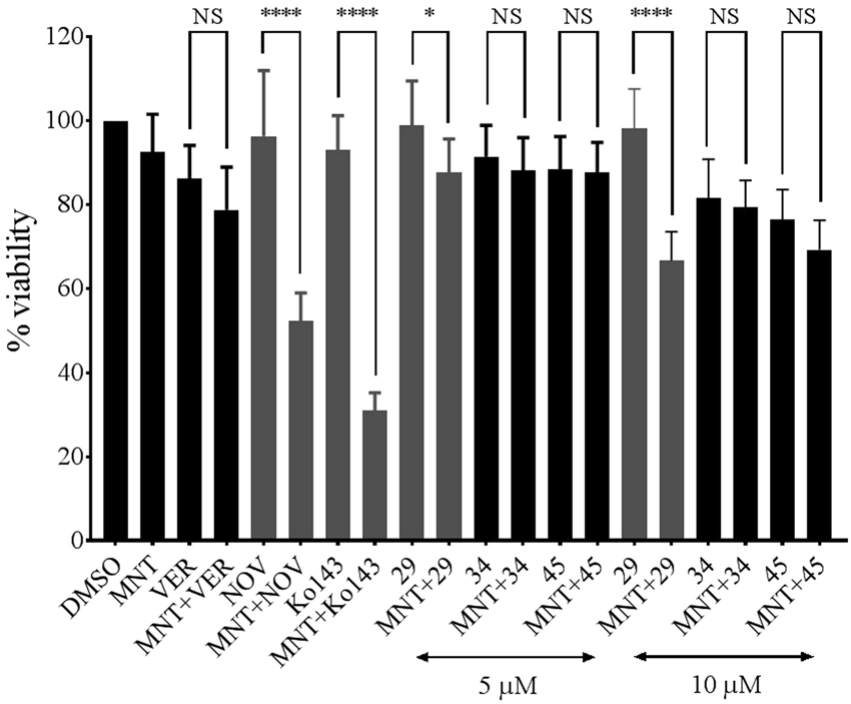
Inhibitors 34 and 45 are specific for P-gp while compound 29 also inhibits BCRP. MCF-7 M100 cells were treated with 50 nM of the chemotherapeutic mitoxantrone and either compound 29, 34, or 45 at 5 or 10 µM, verapamil at 10 µM, novobiocin at 200 µM or Ko143 at 1 µM as indicated in the figure. Each histogram presents the average ± S.D. of the determinations (n = 8, replicates from two individual experiments; ****P < 0.0001; *P < 0.1). MNT, mitoxantrone; VER, verapamil; NOV, novobiocin.

Figure 9 shows the results of experiments that suggest that compounds 34 and 45 inhibit only P-gp, while compound 29 inhibits both P-gp and BCRP. In these experiments, the MCF-7 M100 cells were exposed to mitoxantrone (a BCRP substrate), in the presence or absence of verapamil (a P-gp substrate), novobiocin or Ko143 (BCRP inhibitors), and the P-gp inhibitors 29, 34 or 45 as indicated in the figure. Cell viability was assessed using MTT assays^50,51^. The results indicate that cellular viability of the MCF-7 M100 cell line was reduced when mitoxantrone was co-administered with the BCRP inhibitors, novobiocin and Ko143, but no effect was observed when the P-pg substrate, verapamil, was added. The addition of mitoxantrone or the targeted inhibitors individually did not affect cell viability. When co-administered with mitoxantrone, compounds 34 or 45 did not significantly affect the viability of MCF-7 M100 cells. Compound 29, however, in combination with mitoxantrone, caused statistically significant reduction in cell viability when compared to the viability of the cells in the presence of 29 alone. These results suggest that at the concentrations used, compounds 34 and 45 are P-gp specific, while compound 29 inhibits both P-gp and BCRP.

## Discussion

While chemotherapy resistances may have a variety of causes, multidrug resistance is often caused by the overexpression of P-gp or closely related members of the ABC-transporter family^52,53^. Unfortunately, to date none of the P-gp inhibitors previously identified and pursued as potential co-therapeutics for treatment of MDR diseases has been successful in clinical trials^21,22^. The most recent trials employing the P-gp inhibitor, tariquidar, suffered from toxicities and several Phase III trials have been abandoned, see^47^. A Phase III trial with another P-gp inhibitor, zosuquidar^54^, was recently completed, but did not show improved outcomes in older acute myeloid leukemia patients. The authors of the study speculated that resistances unrelated to P-gp were the cause for the negative outcomes. Despite these set-backs, the potential of P-gp inhibition to improve cancer patient outcomes was demonstrated in an earlier randomized Phase III trial with poor-risk, acute myeloid leukemia patients^23^. These data indicated that positive effects from inhibitors were most prominent in patients with increased P-gp expression. Despite limited success in the clinic, P-gp still seems an important and relevant target for drug discovery and development. More recent advances in the knowledge of the structure and mechanism of P-gp as described in^14,27^ as well as the related drug pumps^55,56^ will likely help discovery of specific inhibitors of specific pumps in a more rational way. Many of the inhibitors previously pursued are thought to bind to the drug binding domains and inhibit chemotherapeutic export by functioning as P-gp transport substrates and competing for transport cycles^22^. This characteristic likely led to the need for relatively high systemic concentrations causing off target toxicities.

In previous work^28^, our group hypothesized that molecules that strongly interacted with the ATP binding domains of P-gp and interfered with the power harvesting of the pump, but that do not bind well to the drug binding domains, would make better leads for inhibiting P-gp. Ultra-high-throughput computational techniques led to the discovery of a number of small, drug-like compounds that were predicted to inhibit P-gp action by specifically interacting with ATP binding and/or hydrolysis^27,28^. Three of these compounds 29, 34 and 45 reversed multidrug resistance in MDR prostate cancer cells in culture^29^ (see Fig. 1 for the chemical structures). Here we described results that strongly suggest that the P-gp inhibitors 29, 34 and 45 reverse multidrug resistance and increase cell mortality in the presence of chemotherapeutics in different cancer cells, in both 2- and 3-dimensional cultures. We showed that these effects are caused by P-gp specific inhibition leading to increased accumulation of P-gp substrates. The decreased P-gp transport activity was shown not to be the result of downregulation of P-gp expression. We also showed that the inhibitors are not transport substrates of P-gp and that compounds 34 and 45 are P-gp specific, whereas compound 29 also affected the activity of the breast cancer resistance protein.

To establish that the effects of the *in silico* identified inhibitors were not cancer-type specific, we extended our studies here to an ovarian cancer cell line, A2780 and its MDR subline, A2780ADR^32,57^. Supplemental Fig. 1 and Table 1 show that the ADR subline exhibited a chemotherapy resistance phenotype to both vinblastine and paclitaxel and that P-gp was significantly overexpressed. We also demonstrated that inclusion of the P-gp inhibitors 29, 34 or 45 with either paclitaxel or vinblastine reversed the multidrug resistance phenotype exhibited by the A2780ADR cells (Fig. 1).

The enzymatic reduction of resazurin (Alamar Blue)^58^ or tetrazolium salts like MTT^50^ are frequently used in medium to high throughput assays to evaluate toxicity of experimental compounds. Both assays respond to mitochondrial metabolism, which is correlated to cell viability, but may not be directly correlated to cell proliferation^59,60^. Figure 1 shows that even at high concentrations of the chemotherapeutic, paclitaxel, significant mitochondrial metabolic activity was observed. As seen in supplemental Fig. 1, the parental A2780 strain showed about 20% residual “metabolic viability” at 10 nM paclitaxel, while more than 50% viability was observed for the A2780ADR line at paclitaxel concentrations 4 orders of magnitude higher. In contrast, vinblastine resulted in residual viability of less than 10% for the parental and ~20% for the MDR line. Similarly, when inhibitors were co-administered with vinblastine, reduction of viability to about 20% was observed. Paclitaxel under these conditions led to about 50% reduction of viability. Both paclitaxel and vinblastine affect microtubule formation and stability, however their mechanisms of action differ. While taxols stabilize microtubules, vinca alkaloids inhibit microtubule polymerization^61–63^. These differences in action may cause different onsets of cell death and may result in the observed prolonged mitochondrial activities.

A main goal of using chemotherapeutics is to induce apoptosis in cancer cells. Paclitaxel stabilizes microtubules leading to G2/M cell cycle arrest and apoptotic cell death^64^. Induction of apoptosis is relatively easy to demonstrate^65^. We showed in Fig. 2 that co-treatment with paclitaxel and P-gp inhibitors significantly induced apoptosis-related characteristics in these MDR cancer cells and increased the number of observed dead cells indicating that inhibition of P-gp resulted in paclitaxel-induced apoptosis. This hypothesis was further supported by the results of colony formation assays using both MDR prostate and ovarian cancer cell lines (Fig. 3). These results taken together strongly suggest that the P-gp inhibitors studied here potentiate the effect of chemotherapeutics on multidrug resistant cancer cells.

While the inhibition of cell proliferation of cancer cells is extremely important, arguably of equal concern is the potential migration of cells to other sites in the body even when proliferation is inhibited. In order to test the effects of P-glycoprotein inhibition on MDR cancer cell migration, a series of wound healing assays were performed. As described in^40–42^, when a nearly confluent two-dimensional cell culture is physically damaged by scratching through the cell layer, migration of cells into the gap can be differentiated from filling the gap by proliferative mechanisms, if the culturing media lacks essential factors required for proliferation. Media with low serum concentrations allow only minimal cell proliferation. Under these conditions, the “healing” of the wound can occur only by the migration of existing cells into the scratch zone. In experiments like these, filling of the gap with cells (“healing or wound closure”) likely occurs by detachment and movement of preexisting cells into the gap^40–42^. Our results (Fig. 4) strongly indicated that the presence of the P-gp inhibitors 29, 34, or 45 with vinblastine inhibited wound closure by 30 to 50% suggesting that an increased accumulation of vinblastine in cells treated with P-gp inhibitors inhibited cell migration. Vinblastine is known to interact with tubulin, resulting in perturbations of microtubule polymerization^66^ which is a necessary requirement for cellular mobility.

Increased intracellular accumulation of P-gp substrates, calcein AM and daunorubicin, upon inhibition of P-gp activity by our experimental compounds was then shown in Fig. 5. Calcein AM is frequently used as a probe to test P-gp and MRP-1 function in cultured cell lines, as well as in primary cancer cells from patients for treatment outcome predictions^67–69^. Calcein AM is a transport substrate for P-gp, while the hydrolysis product, calcein, is not transported by the pump. High P-gp activities can therefore be correlated with the lack of intrinsic fluorescence of calcein, while inhibition of P-gp or lack of expression can be correlated with increased intracellular fluorescence. We demonstrated that calcein AM accumulation in the MDR ovarian cancer cell line significantly increased in the presence of the P-gp inhibitors. Very similar increases in accumulation of daunorubicin in the presence of P-gp specific inhibitors 29, 34 and 45 were also demonstrated. These results are consistent with 29, 34 and 45 blocking P-gp catalyzed export of calcein AM or daunorubicin, causing cellular accumulation of the substrates. Control experiments suggested that these effects were due to inhibition of P-glycoprotein and not due to the activities of BCRP or MRP-1.

One of the potential factors that negatively affected success in clinical trials of P-gp inhibitors was lack of tumor penetration of some of the inhibitors^70,71^. To evaluate whether the experimental P-gp inhibitors facilitated P-gp inhibition in what might be more of a clinically relevant 3-dimensional culture, microtumor spheroids were grown using the multidrug resistant DU145TXR prostate cancer cell line. Cellular accumulation of P-gp substrates as well as cytotoxicity of chemotherapeutics were then assessed. In attempts to create spheroids from the MDR ovarian cancer cell line, A2780ADR, we observed a lack of tight association of the cells that was likely due to low expression of claudin 4 which is required for spheroid formation^72^. The MDR prostate cancer cell line, DU145TXR, however, showed relatively tight cell to cell association in the microtumors produced. Results of experiments using these microtumors treated with P-gp inhibitors showed stronger accumulation of calcein AM also in internal parts of the spheroid (Fig. 6). In time course experiments, nearly complete tumor penetration of the fluorescent dye was observed after about 100 minutes of incubation, suggesting that the P-gp inhibitors affected cells inside the tumor. Increased cytotoxicity of daunorubicin in the presence of inhibitor 29 indicated that the inclusion of the P-gp inhibitor resulted in significantly increased cell mortality and destruction of these microtumors (Fig. 7).

It had been previously shown by others that chemotherapeutics or P-glycoprotein inhibitors affected P-gp protein expression levels in cancer cells and that decreased expression of P-gp can be a cause of re-sensitization of MDR cells. Cisplatin, for example, has been observed to increase P-gp expression^73,74^, while agents like curcumin or certain antipsychotics appear to decrease P-gp expression^44,45^. We evaluated the effects of the targeted P-gp inhibitors on P-gp expression using Western blot analyses and found that exposure of the cancer cells to the P-gp inhibitors 29, 34 and 45 did not result in decreases in P-glycoprotein expression. This suggests that the effects presented here were the result of direct inhibition of the transport activities of the pump by compounds 29, 34 and 45, and were not related to changes in protein expression.

The original premise and hypothesis for our studies was that P-gp inhibitors that are not transport substrates by themselves will make better drug leads for future development^28,29^. We presented biochemical data in^28^ that supported the hypothesis that inhibitors 29, 34 and 45 were not transport substrates. Here we presented strong evidence that these inhibitors are not transported by P-gp, since the presence of the strong P-gp inhibitor, tariquidar, did not significantly affect accumulation of the compounds (Fig. 8).

Additional experiments were performed to assess the specificity of inhibition by compounds 29, 34 and 45 on a second ABC transporter, BCRP, using a BCRP overexpressing MDR breast cancer cell line (Fig. 9). These experiments indicated that the P-gp inhibitors 34 and 45 did not significantly resensitize the BCRP overexpressing cell line to mitoxantrone, which is a BCRP substrate and chemotherapeutic. This suggests that the P-gp inhibitors 34 and 45 discriminated between the two closely related ABC transporters, P-glycoprotein and BCRP. Compound 29 on the other hand did resensitize the BCRP overexpressing cell line to mitoxantrone, indicating it did not discriminate between the two transporters. It will be of interest in the future to extend these investigations of the discrimination of the P-gp inhibitors to other ABC transporters and more generally to other ATP-utilizing enzymes.

P-glycoprotein has been a target for drug discovery for almost 40 years (for reviews see^20–22^). Other investigators also describe P-gp inhibitors that interact with the nucleotide binding domains of P-gp^75,76^ although translation into the clinic has not yet been reported. Others recently reported *in silico* drug discovery studies^77^. However, these studies targeted the drug binding domains in attempts to disrupt drug transport by P-gp. This approach may lead to P-gp inhibitors that are transported by P-gp, a characteristic that we have tried to avoid.

## Conclusions

We have shown here that the targeted inhibitors that were initially discovered through computational high throughput drug docking studies specifically inhibited P-glycoprotein function and increased the accumulation of P-gp transport substrates in 2- and 3-dimensional cell cultures of chemotherapy resistant ovarian and prostate cancer cell lines. We showed that the inhibitors were not themselves P-gp transport substrates, but increased accumulation of chemotherapeutics and caused reduction of cell viability, reduced colony formation, reduced cell migration, and increased cell death in both 2- and 3-dimensional cell culture studies. The reversal of the multidrug resistance phenotypes observed here was shown not to be due to downregulation of P-gp expression. P-gp inhibitors 34 and 45 did not appear to affect BCRP transport activities, while compound 29 affected both P-gp and BCRP. The experimental compounds 29, 34 and 45 therefore appear to be promising candidates for further development into co-therapeutics to treat cancers that are multidrug resistant due to P-gp overexpression.

## Materials and Methods

### Cell lines and cell culture

The chemotherapeutic sensitive DU145 human prostate cancer cells^78^ as well as the multidrug resistant sub-line, DU145TXR^30^ were generous gifts from Dr. Evan Keller (University of Michigan, Ann Arbor, MI). The MDR DU145TXR was maintained under positive selection pressure by supplementing complete medium with 10 nM paclitaxel (Acros Organics, NJ). The above mentioned cell lines as well as the chemotherapeutic sensitive A2780 ovarian cancer cells (93112519, Sigma) and the multidrug resistant A2780ADR (93112520, Sigma)^32,57^ were maintained in complete media consisting of RPMI-1640 with L-glutamine, 10% fetal bovine serum (FBS; BioWest, Logan, UT), 100 U/mL penicillin and 100 μg/mL streptomycin in a humidified incubator at 37 °C and 5% CO_2_. The drug-resistant line A2780ADR was maintained under positive selection pressure by supplementing complete medium with 100 nM doxorubicin (Fisher Scientific, NJ). Cell culture materials were purchased from Corning Inc. (Corning, NY) unless otherwise stated. A BCRP over-expressing breast cancer cell line (MCF-7 M100) was established by us according to a previously described method^49^. The drug sensitive MCF-7 (ATCC)^48^ breast cancer cell line was exposed to increasing concentrations of the chemotherapeutic, mitoxantrone, over 60 passages. The mitoxantrone resistant MCF-7 M100 cell line was maintained under positive selection pressure by supplementing complete medium with 100 nM mitoxantrone (Santa Cruz Biotechnology, CA).

### Western blot analyses

Whole cell lysates were prepared using approximately five million cells from each cell line. Cells were lysed in 500 μL of SDS buffer (125 mM Tris HCl pH 6.8, 20% v/v glycerol, 4% w/v SDS and 2% v/v β-mercaptoethanol) containing 5 μL of protease inhibitor cocktail (P8340, Sigma). The lysates were filtered through a spin column (QIAprep ®) by centrifugation at 5000 rpm for 5 minutes and used for Western blot analysis. The lysate proteins were resolved by denaturing SDS-PAGE^79^ for 100 minutes at 110 V and subsequently transferred to a PVDF membrane (Bio-Rad, CA) using a Mini Transblot cell (Bio-Rad) for 70 minutes at 110 V. The transfer buffer contained 192 mM glycine, 25 mM Tris, and 10% methanol. The membrane was blocked overnight at 4 °C with 4% powdered skimmed milk in TBS-T (12 mM Tris–HCl pH 7.5, 0.5 M NaCl, 0.05% Tween 20). Washed membranes were incubated with the P-gp mouse monoclonal antibody C219 (Enzo Life Sciences, NY), the BCRP-specific monoclonal antibody B1 (from Santa Cruz Biotechnology, CA), or the β-actin monoclonal antibody C4 (Santa Cruz Biotechnology, CA), diluted to between 1:500 and 1:2000 in TBS-T and 4% powdered skimmed milk for 2 hours at room temperature. Washed membranes were subsequently incubated for 1 hour at room temperature with alkaline horseradish peroxidase-conjugated goat anti-mouse secondary antibody sc-2005 (Santa Cruz Biotechnology, CA) diluted to 1:10000 in TBS-T containing 4% milk powder. Membranes were washed in TBS-T and P-gp, BCRP, or β-actin were visualized using enhanced chemiluminescence detection (ECL kit, Thermo Scientific, IL). To evaluate P-gp protein expression levels of cells after inhibitor treatment, DU145TXR cells were treated with 5 μM of P-gp inhibitors 29, 34 or 45 for 48 hours after which cell lysates were prepared and Western blot analyses were performed as described above.

### Resazurin cell viability assay

The resazurin assay is a well-established cell viability assay^80^ which relies on the reduction of the blue, water soluble resazurin to highly fluorescent resafurin^80^ under the reducing environment in the cell. The fluorescence of resafurin is directly proportional to the number of viable cells and can be measured by excitation at 530 nm and emission at 590 nm^80^. The assay was performed as follows: Cells were trypsinized from monolayers and seeded with 4000 cells in 150 μL of complete medium in a 96 well plate. After 24 hours, cells were treated with chemotherapeutics and / or P-gp inhibitory compounds dissolved in DMSO, or DMSO controls diluted in complete medium for 48 hours. The chemotherapeutics, paclitaxel and vinblastine, were purchased from Acros Organics, NJ, and MP Biomedicals, France, respectively. Upon 42 hours of treatment, resazurin assays were performed as described in^33^ using 440 μM of resazurin (Acros Organics, NJ) solution prepared in PBS (137 mM NaCl, 2.7 mM KCl, 10 mM Na_2_HPO_4_, 1.8 mM KH_2_PO_4_, pH 7.4). After 6 hours of incubation with resazurin, the resulting fluorescence was measured by excitation at 530 nm and emission at 590 nm using a Bio-Tek Synergy 2 multi-mode plate reader (Bio-Tek, Winooski, VT). DMSO was used as the vehicle, 250 μM probenecid and 60 μM novobiocin (both from Alfar Aesar, MA) were used as negative controls, and verapamil (MP Biomedicals, France) was used as a positive control for P-gp inhibition. Percent viability was calculated using DMSO treated cells as representative for 100% viability. Background fluorescence was determined using resazurin and complete medium without cells.

1 $${\rm{ \% }}\,{\rm{V}}{\rm{i}}{\rm{a}}{\rm{b}}{\rm{i}}{\rm{l}}{\rm{i}}{\rm{t}}{\rm{y}}\,=100\ast \frac{{\rm{F}}{\rm{l}}{\rm{u}}{\rm{o}}{\rm{r}}{\rm{e}}{\rm{s}}{\rm{c}}{\rm{e}}{\rm{n}}{\rm{c}}{\rm{e}}\,{\rm{o}}{\rm{f}}\,{\rm{e}}{\rm{x}}{\rm{p}}{\rm{e}}{\rm{r}}{\rm{i}}{\rm{m}}{\rm{e}}{\rm{n}}{\rm{t}}{\rm{a}}{\rm{l}}\,{\rm{c}}{\rm{e}}{\rm{l}}{\rm{l}}{\rm{s}}\,-\,{\rm{B}}{\rm{a}}{\rm{c}}{\rm{k}}{\rm{g}}{\rm{r}}{\rm{o}}{\rm{u}}{\rm{n}}{\rm{d}}\,{\rm{f}}{\rm{l}}{\rm{u}}{\rm{o}}{\rm{r}}{\rm{e}}{\rm{s}}{\rm{c}}{\rm{e}}{\rm{n}}{\rm{c}}{\rm{e}}}{{\rm{F}}{\rm{l}}{\rm{u}}{\rm{o}}{\rm{r}}{\rm{e}}{\rm{s}}{\rm{c}}{\rm{e}}{\rm{n}}{\rm{c}}{\rm{e}}\,{\rm{o}}{\rm{f}}\,{\rm{D}}{\rm{M}}{\rm{S}}{\rm{O}}\,{\rm{t}}{\rm{r}}{\rm{e}}{\rm{a}}{\rm{t}}{\rm{e}}{\rm{d}}\,{\rm{c}}{\rm{e}}{\rm{l}}{\rm{l}}{\rm{s}}\,-\,{\rm{B}}{\rm{a}}{\rm{c}}{\rm{k}}{\rm{g}}{\rm{r}}{\rm{o}}{\rm{u}}{\rm{n}}{\rm{d}}\,{\rm{f}}{\rm{l}}{\rm{u}}{\rm{o}}{\rm{r}}{\rm{e}}{\rm{s}}{\rm{c}}{\rm{e}}{\rm{n}}{\rm{c}}{\rm{e}}}$$

The results were plotted as the mean with standard deviation (SD) of twelve replicates per concentration from at least two independent experiments. The graphical representations and IC_50_ values were determined using four parameter variable slope non-linear regression, using the following equation: Y=bottom + (top-bottom)/(1 + 10^((logIC50-X)*Hill Slope) (GraphPad Prism™, La Jolla California, USA, Version 6.05). The reported “fold sensitization” was calculated as follows.

2 $${\rm{F}}{\rm{o}}{\rm{l}}{\rm{d}}\,{\rm{s}}{\rm{e}}{\rm{n}}{\rm{s}}{\rm{i}}{\rm{t}}{\rm{i}}{\rm{z}}{\rm{a}}{\rm{t}}{\rm{i}}{\rm{o}}{\rm{n}}=\frac{{{\rm{I}}{\rm{C}}}_{50}\,{\rm{v}}{\rm{a}}{\rm{l}}{\rm{u}}{\rm{e}}\,{\rm{o}}{\rm{f}}\,{\rm{A}}2780{\rm{A}}{\rm{D}}{\rm{R}}\,{\rm{c}}{\rm{e}}{\rm{l}}{\rm{l}}{\rm{s}}\,{\rm{t}}{\rm{r}}{\rm{e}}{\rm{a}}{\rm{t}}{\rm{e}}{\rm{d}}\,{\rm{w}}{\rm{i}}{\rm{t}}{\rm{h}}\,{\rm{c}}{\rm{h}}{\rm{e}}{\rm{m}}{\rm{o}}{\rm{t}}{\rm{h}}{\rm{e}}{\rm{r}}{\rm{a}}{\rm{p}}{\rm{e}}{\rm{u}}{\rm{t}}{\rm{i}}{\rm{c}}\,{\rm{o}}{\rm{n}}{\rm{l}}{\rm{y}}}{\begin{array}{c}{{\rm{I}}{\rm{C}}}_{50}\,{\rm{v}}{\rm{a}}{\rm{l}}{\rm{u}}{\rm{e}}\,{\rm{o}}{\rm{f}}\,{\rm{A}}2780{\rm{A}}{\rm{D}}{\rm{R}}\,{\rm{o}}{\rm{r}}\,{\rm{A}}2780\,{\rm{c}}{\rm{e}}{\rm{l}}{\rm{l}}{\rm{s}}\,{\rm{t}}{\rm{r}}{\rm{e}}{\rm{a}}{\rm{t}}{\rm{e}}{\rm{d}}\,{\rm{w}}{\rm{i}}{\rm{t}}{\rm{h}}\,{\rm{c}}{\rm{h}}{\rm{e}}{\rm{m}}{\rm{o}}{\rm{t}}{\rm{h}}{\rm{e}}{\rm{r}}{\rm{a}}{\rm{p}}{\rm{e}}{\rm{u}}{\rm{t}}{\rm{i}}{\rm{c}}\\ {\rm{a}}{\rm{n}}{\rm{d}}\,{\rm{P}}{\rm{g}}{\rm{p}}\,{\rm{i}}{\rm{n}}{\rm{h}}{\rm{i}}{\rm{b}}{\rm{i}}{\rm{t}}{\rm{o}}{\rm{r}}{\rm{y}}\,{\rm{c}}{\rm{o}}{\rm{m}}{\rm{p}}{\rm{o}}{\rm{u}}{\rm{n}}{\rm{d}}\end{array}}$$

### MTT cell viability assay for BCRP over-expressing MCF-7 M100 breast cancer cell line

MCF-7 M100 cells were trypsinized from monolayers and seeded with 2500 cells in 150 μL of complete medium in a 96 well plate. After 24 hours, cells were treated with mitoxantrone (50 nM, Santa Cruz Biotechnology, CA) and / or P-gp inhibitory compounds dissolved in DMSO, or DMSO controls diluted in complete medium for 96 hours. After 96 hours of treatment, MTT assays were performed as described in^81^ using 5 mg/mL of MTT (Acros Organics, NJ) solution prepared in PBS (137 mM NaCl, 2.7 mM KCl, 10 mM Na_2_HPO_4_, 1.8 mM KH_2_PO_4_, pH 7.4). After 4 hours of incubation with MTT, the media was removed and the formazan crystals were dissolved in 100 µL of DMSO. The absorbance at 570 nm was then measured using a BioTek Cytation 5 imaging multi-mode reader (Bio-Tek, Winooski, VT). Data were obtained from two independent experiments. DMSO was used as the vehicle, 200 μM of novobiocin (Alfar Aesar, MA) and 1 μM of Ko143 (Sigma) were used as positive controls, and verapamil (MP Biomedicals, France) was used as a negative control for BCRP inhibition. Percent viability was calculated using DMSO treated cells as representative for 100% viability.

### Colony formation assay

Colony formation assays were performed similar to those described in^39^ with slight modifications. A2780ADR cells were seeded at 4000 cells per well in 96 well plates for 24 hours and incubated for 48 hours with chemotherapeutics vinblastine (0.1 µM**)** or paclitaxel (1 µM) alone, as well as 10 µM inhibitors, compounds 29, 34, 45 or verapamil alone, or combinations of chemotherapeutics and inhibitors at the concentrations given above. After 48 hours, the media were replaced with drug free media and cells were allowed to grow for an additional 96 hours. To visualize cells that had grown during that period, media were removed from the wells of 96 well plates and cells were fixed with a mixture of methanol and acetic acid 3:1 (v/v) solution. After 5 minutes, the fixation solution was removed and the cells were stained with 0.5% w/v crystal violet (Alfar Aesar, MA) in 25% methanol for 30 minutes. Finally, crystal violet was removed and the plates were washed with running water to remove excess crystal violet. Cells that had continued to grow over the 96 hour incubation time were visible as blue dots in nearly confluent cell colonies. No growth was observed where P-gp inhibitors and chemotherapeutic were co-administered during the initial 48 hr incubation.

In a more quantitative colony formation assay similar to^39^, DU145 TXR cells were seeded in 24 well plates with 200 cells per well. After 24 hours, cells were treated with 500 nM paclitaxel or 5 μM inhibitors alone, as well as combinations of inhibitors and chemotherapeutics for 48 hours. The media was then removed and cells were allowed to form colonies for 5 days in drug-free complete media. Cells were fixed and stained as described above. Colonies visible to the naked eye were counted and recorded by persons blinded to all experimental conditions. The experiment was repeated two times.

### Scratch assay

Scratch assays were performed as outlined previously^41^ with minor modifications. Cells were trypsinized from monolayers and diluted in complete culture medium to a density of 25,000 cells in 300 μL cell suspension per well in 48-well plates and cultured until confluent. The monolayers of cells were scratched using a 200 μL pipette tip. Media was removed and the cells were washed with PBS to remove any floating cells. Low serum (1%) containing media was then added to the wells together with 0.1 μM vinblastine with or without 2.5 or 5 µM P-gp inhibitory compounds, or 0.5% DMSO as a drug-carrier vehicle control. Immediately after the scratching and media additions, the wounds were imaged using a BioTek Cytation 5 imaging multi-mode reader (Bio-Tek, Winooski, VT). After 14 hours, the remaining wounds were imaged again and the areas of the wounds before and after treatment were quantified using ImageJ software^82^. The percentage of wound closures in each test were calculated compared to vehicle treated experiments. Each individual experiment was performed in triplicate and 2 images were obtained for each well. The whole experiment was repeated at least once, and n = 12 was used for the statistical analysis.

### Calcein AM assay

To assess inhibition of P-gp-catalyzed transport of the P-gp pump substrate, calcein AM, A2780ADR cells were seeded at 40,000 cells per well in 96 wells plates and allowed to grow in complete medium for 48 hours. Medium was removed and cells were treated with or without 10 μM P-gp inhibitory compounds and 2.5 μM calcein AM (Life Technologies, OR) and diluted into phenol red free RPMI 1640 media. The cells were imaged over 45 minutes in 15 minute intervals using both GFP fluorescence and bright field filters. Fluorescence was measured by excitation at 485 nm with a 20 nm gate and at emission at 535 nm with a 20 nm gate using a BioTek Cytation 5 imaging multi-mode reader (Bio-Tek, Winooski, VT). DMSO was used as the vehicle, 10 μM experimental compound 19 (which does not penetrate intact cells)^29^, 250 μM probenecid and 60 μM novobiocin were used as negative controls, and verapamil was used as a positive control for competitive inhibition of P-gp transport. Results were plotted as the mean with standard deviation (SD) of three replicates per concentration and are representative of at least two independent experiments.

### Daunorubicin accumulation experiments

A2780ADR cells were seeded in 96 wells plates at 150,000 cells per well in complete media and allowed to grow overnight. Medium was then removed and cells were treated with or without 15 μM P-gp inhibitory compounds and in the presence of 10 μM daunorubicin (MP Biomedicals, France) diluted in complete medium. After 2.5 hours of incubation, media were removed and cells were washed once with PBS containing DMSO 5% and once with 2% DMSO and imaged using a Texas Red fluorescence filter and a BioTek Cytation 5 imaging multi-mode reader.

To quantify the accumulation of daunorubicin in cells, assays were carried out as above, but cells were lysed in 100 μL of PBS containing 0.5% SDS and 0.5% Triton X100 immediately after the washing step. The fluorescence of daunorubicin was measured using excitation at 488 nm with a 20 nm gate and emission at 575 nm with a 20 nm gate using the BioTek Cytation 5 imaging multi-mode reader.

### Calcein AM uptake in spheroids

Spheroids of the multidrug resistant prostate cancer cell line, DU145TXR, were produced as described^83^ with the following modifications. Cells were trypsinized from monolayers and diluted in complete culture medium to a density of 15000 cells in a 200 μL cell suspension per well in 96-well plates. Prior to the experiment, all wells used for the assay had been coated with 2.5% low melting agarose in RPMI. After seeding, the 96-well plates were centrifuged at 600 rpm for 20 minutes. Centrifugation was repeated after 24 hours to obtain more tightly packed spheroids. Spheroids that had formed six days after seeding were used for experiments. Spheroids were pretreated with 15 µM of P-gp inhibitors 29, 34 or 45 for 3 hours and then incubated with 2.5 µM of calcein-AM. Fluorescence images were obtained every 20 minutes for a total of 100 minutes using a GFP filter. The resulting TIFF image files were analyzed using ImageJ software and interactive 3D surface plot plugging was used to obtain 3D graphs based on the pixel intensities of images. Each experiment was carried out in triplicate and the whole experiment was duplicated.

### Spheroid growth and spheroid area reduction assay

Spheroid cultures were prepared as described above except that the growth was initiated with only 2000 cells per spheroid. Prepared plates were incubated at 37 °C for 72 hours in a humidified incubator with 5% CO_2_ for spheroid formation. The spheroids were treated with daunorubicin at the concentrations indicated, with or without compound 29 that had been diluted in 50 μL of complete medium. Half of the medium was replaced after every 48 hours of incubation. The spheroids were imaged every 24 hours using a BioTek Cytation 5 imaging multi-mode reader and the areas of the spheroids were determined using the BioTek Gen5 software. The fold change of tumor spheroid area was determined each day by comparing the area of each spheroid to that of day one. Dead cells in the spheroid culture on day six were visualized and imaged after staining with 10 μL of a 0.01% ethidium bromide (Fisher Scientific, NJ) solution diluted into PBS.

### Fluorescence microscopic analysis of cell apoptosis

Double staining with acridine orange/ ethidium bromide (AO/EB) is a reliable method to detect apoptosis and was carried out as described in^37^ with slight modifications. Briefly, 16000 cells were seeded in 48 well plates in 300 μL of complete media and incubated for 24 hours. After 24 hours, cells were treated with 1 μM paclitaxel and 10 μM P-gp inhibitory compounds in DMSO or DMSO controls for 48 hours. Then dual stain containing solution AO/EB (100 μg/ml each) was added to each well and images were acquired using a BioTek Cytation 5 imaging multi-mode reader with GFP (for green fluorescence from acridine orange), Texas Red fluorescence (for red fluorescence from ethidium bromide) and bright field filters.

### Cellular Accumulation Assays for Experimental P-gp Inhibitors

DU145TXR cells were seeded in 6 well plates with ~350,000 cells per well. After 48 hours, the media was replaced with fresh media and cells were treated with 5 μM of compounds (29, 34, or 45) and daunorubicin with or without 500 nM of tariquidar (MedKoo Biosciences, Chapel Hill, NC, U.S.A.) Experiments were performed in triplicate. After 2.5 hours of incubation with compounds, cells were washed with Hank’s Balanced Salt Solution (HBSS, Corning Inc. NY), harvested using trypsin, and counted. Each sample was then washed with 2 mL of ice-cold HBSS and diluted in cold HBSS at a final concentration of 1 million cells/mL. All samples were frozen with liquid nitrogen and stored at −80 °C until analysis. LC-MS/MS analyses were performed essentially as described in^84^. 250 µl of treated or untreated cell lysate was aliquoted into Eppendorf tubes. Blank lysates were spiked with varying concentrations of each compound to create a standard curve. Each sample was mixed with 0.5 ml of a solution containing 0.15% formic acid and 120 ng/ml n-benzylbenzamide internal standard in methanol, vortexed 15 sec, incubated 10 min at RT and then centrifuged twice at 16,100 × g. The supernatant was then analyzed by LC-MS/MS using a Sciex 4000QTRAP mass spectrometer coupled to a Shimadzu Prominence LC. Chromatography conditions were as follows. Buffer A consisted of water + 0.1% formic acid and Buffer B consisted of methanol + 0.1% formic acid for compound 29, 34, and 45 and acetonitrile + 0.1% formic acid for daunorubicin. The column flow rate was 1.5 ml/min using an Agilent C18 XDB, 5 micron packing 50 × 4.6 mm column. The gradient conditions for compounds 29, 34, and 45 were 0–1.0 min 3% B, 1.0–2.0 min gradient to 95% B, 2.0–3.5 min 95% B, 3.5–3.6 min gradient to 3% B. 3.6–4.5 min 3% B. Gradient conditions for daunorubicin were 0–2.0 min 5% B, 2.0–3.5 min gradient to 60% B, 3.5–5.0 min 60% B, 5.0–5.1 min gradient to 5% B, 5.1–7.5 min 5% B. Compounds were detected in MRM mode after optimization of machine parameters by detection of the following parent/daughter ions: 459.1/278.1 for 29, 477.1/285.1 for 34, 424.1/149.0 for 45, and 528.1/321.0 for daunorubicin. N-benzyl benzamide (212.1/91.1) was used as the internal standard. A value 3-fold above the signal obtained from blank lysate was designated as the limit of detection (LOD). The limit of quantitation (LOQ) was defined as the lowest concentration of standard at which back calculation yielded a concentration within 20% of theoretical and above the LOD. The LOQ for all analytes was between 0.1–0.5 ng/ml. The protein pellet remaining after addition of organic solvent was resuspended in 25 µl of 0.1 M NaOH, boiled for 5 min, and 5 µl was mixed with 200 µl of 1:50 B:A reagent (Thermofisher BCA Kit) in order to determine the protein concentration. A BSA standard curve was prepared in H_2_O and mixed in the same ratio. The samples were incubated 30 min at 37 °C and read at 562 nM. Compound concentrations in the lysates were then normalized to protein content for each sample.

### Data availability

The datasets generated during and/or analyzed during the current study are available from the corresponding authors on reasonable request.

## Electronic supplementary material

Supplementary Information

## References

[1] Zhao J (2016). Cancer stem cells and chemoresistance: The smartest survives the raid. Pharmacol Ther.

[2] Wijdeven RH, Pang B, Assaraf YG, Neefjes J (2016). Old drugs, novel ways out: Drug resistance toward cytotoxic chemotherapeutics. Drug Resist Updat.

[3] Sun Y (2016). Tumor microenvironment and cancer therapy resistance. Cancer Lett.

[4] Fruci D, Cho WC, Nobili V, Locatelli F, Alisi A (2016). Drug Transporters and Multiple Drug Resistance in Pediatric Solid Tumors. Current drug metabolism.

[5] Gottesman MM, Pastan I (1993). Biochemistry of multidrug resistance mediated by the multidrug transporter. Annu Rev Biochem.

[6] Ferreira RJ, dos Santos DJ, Ferreira MJ (2015). P-glycoprotein and membrane roles in multidrug resistance. Future Med Chem.

[7] Chen CJ (1986). Internal duplication and homology with bacterial transport proteins in themdr1 (P-glycoprotein) gene from multidrug-resistant human cells. Cell.

[8] Higgins CF (2007). Multiple molecular mechanisms for multidrug resistance transporters. Nature.

[9] Cascorbi, I. P-glycoprotein: tissue distribution, substrates, and functional consequences of genetic variations. *Handbook of experimental pharmacology*, 261–283 (2011). 10.1007/978-3-642-14541-4_6 21103972

[10] Goldstein LJ (1989). Expression of multidrug resistance gene in human cancers. Journal of the National Cancer Institute.

[11] Mechetner E (1998). Levels of multidrug resistance (MDR1) P-glycoprotein expression by human breast cancer correlate with *in vitro* resistance to taxol and doxorubicin. Clinical Cancer Research.

[12] Penson RT (2004). Expression of multidrug resistance-1 protein inversely correlates with paclitaxel response and survival in ovarian cancer patients: a study in serial samples. Gynecologic oncology.

[13] Sharom FJ (2008). ABC multidrug transporters: structure, function and role in chemoresistance. Pharmacogenomics.

[14] McCormick JW, Vogel PD, Wise JG (2015). Multiple Drug Transport Pathways through Human P-Glycoprotein. Biochemistry.

[15] Fojo A (1987). Molecular biology of drug resistance. Breast Cancer Res Treat.

[16] Fojo AT, Shen DW, Mickley LA, Pastan I, Gottesman MM (1987). Intrinsic drug resistance in human kidney cancer is associated with expression of a human multidrug-resistance gene. Journal of clinical oncology: official journal of the American Society of Clinical Oncology.

[17] Eckford PD, Sharom FJ (2009). ABC efflux pump-based resistance to chemotherapy drugs. Chem Rev.

[18] Gottesman MM (2002). Mechanisms of cancer drug resistance. Annual review of medicine.

[19] Gottesman MM, Fojo T, Bates SE (2002). Multidrug resistance in cancer: role of ATP-dependent transporters. Nature reviews. Cancer.

[20] Kathawala RJ, Gupta P, Ashby CR, Chen Z-S (2015). The modulation of ABC transporter-mediated multidrug resistance in cancer: A review of the past decade. Drug Resistance Updates.

[21] Binkhathlan Z, Lavasanifar A (2013). P-glycoprotein inhibition as a therapeutic approach for overcoming multidrug resistance in cancer: current status and future perspectives. Current cancer drug targets.

[22] Palmeira A, Sousa E, Vasconcelos MH, Pinto MM (2012). Three decades of P-gp inhibitors: skimming through several generations and scaffolds. Curr Med Chem.

[23] List AF (2001). Benefit of cyclosporine modulation of drug resistance in patients with poor-risk acute myeloid leukemia: a Southwest Oncology Group study. Blood.

[24] Szakacs G, Paterson JK, Ludwig JA, Booth-Genthe C, Gottesman MM (2006). Targeting multidrug resistance in cancer. Nature reviews. Drug discovery.

[25] Gottesman, M.M. & Pastan, I.H. The Role of Multidrug Resistance Efflux Pumps in Cancer: Revisiting a JNCI Publication Exploring Expression of the MDR1 (P-glycoprotein) Gene. *J Natl Cancer Inst* **107** (2015). 10.1093/jnci/djv222 PMC4836801 26286731

[26] Thomas H, Coley HM (2003). Overcoming multidrug resistance in cancer: an update on the clinical strategy of inhibiting p-glycoprotein. Cancer control: journal of the Moffitt Cancer Center.

[27] Wise JG (2012). Catalytic transitions in the human MDR1 P-glycoprotein drug binding sites. Biochemistry.

[28] Brewer FK, Follit CA, Vogel PD, Wise JG (2014). In silico Screening for Inhibitors of P-Glycoprotein that Target the Nucleotide Binding Domains. Molecular pharmacology.

[29] Follit CA, Brewer FK, Wise JG, Vogel PD (2015). In silico identified targeted inhibitors of P-glycoprotein overcome multidrug resistance in human cancer cells in culture. Pharmacol Res Perspect.

[30] Takeda M (2007). The establishment of two paclitaxel-resistant prostate cancer cell lines and the mechanisms of paclitaxel resistance with two cell lines. Prostate.

[31] Eva A (1982). Cellular genes analogous to retroviral onc genes are transcribed in human tumour cells. Nature.

[32] Rogan AM, Hamilton TC, Young RC, Klecker RW, Ozols RF (1984). Reversal of adriamycin resistance by verapamil in human ovarian cancer. Science.

[33] Riss, T. L. *et al*. in Assay Guidance Manual. (eds G.S. Sittampalam *et al*.) (Bethesda (MD); 2004).

[34] O’Brien J, Wilson I, Orton T, Pognan F (2000). Investigation of the Alamar Blue (resazurin) fluorescent dye for the assessment of mammalian cell cytotoxicity. Eur J Biochem.

[35] Yang CH, Chen YC, Kuo ML (2003). Novobiocin sensitizes BCRP/MXR/ABCP overexpressing topotecan-resistant human breast carcinoma cells to topotecan and mitoxantrone. Anticancer Res.

[36] Issandou M, Grand-Perret T (2000). Multidrug resistance P-glycoprotein is not involved in cholesterol esterification. Biochem Biophys Res Commun.

[37] Ribble D, Goldstein NB, Norris DA, Shellman YG (2005). A simple technique for quantifying apoptosis in 96-well plates. BMC Biotechnol.

[38] Liu K, Liu PC, Liu R, Wu X (2015). Dual AO/EB staining to detect apoptosis in osteosarcoma cells compared with flow cytometry. Med Sci Monit Basic Res.

[39] Sun Y (2011). Effects of an Indolocarbazole-Derived CDK4 Inhibitor on Breast Cancer Cells. Journal of Cancer.

[40] Evans CP, Elfman F, Cunha G, Shuman MA (1997). Decreased prostate cancer cell migration by inhibition of the insulin-like growth factor II/Mannose-6-Phosphate receptor. Urol Oncol.

[41] Valero, M. L., Mello de Queiroz, F., Stuhmer, W., Viana, F. & Pardo, L. A. TRPM8 ion channels differentially modulate proliferation and cell cycle distribution of normal and cancer prostate cells. *PLoS One* **7**, e51825 (2012). 10.1371/journal.pone.0051825 PMC3522609 23251635

[42] Liang CC, Park AY, Guan JL (2007). *In vitro* scratch assay: a convenient and inexpensive method for analysis of cell migration *in vitro*. Nat Protoc.

[43] Homolya L (1993). Fluorescent cellular indicators are extruded by the multidrug resistance protein. Journal of Biological Chemistry.

[44] Shin SY, Choi BH, Kim JR, Kim JH, Lee YH (2006). Suppression of P-glycoprotein expression by antipsychotics trifluoperazine in adriamycin-resistant L1210 mouse leukemia cells. European journal of pharmaceutical sciences: official journal of the European Federation for Pharmaceutical Sciences.

[45] Choi BH, Kim CG, Lim Y, Shin SY, Lee YH (2008). Curcumin down-regulates the multidrug-resistance mdr1b gene by inhibiting the PI3K/Akt/NF kappa B pathway. Cancer Lett.

[46] Di Nicolantonio F (2004). *Ex vivo* reversal of chemoresistance by tariquidar (XR9576). Anticancer Drugs.

[47] Tamaki A, Ierano C, Szakacs G, Robey RW, Bates SE (2011). The controversial role of ABC transporters in clinical oncology. Essays in biochemistry.

[48] Soule HD, Vazguez J, Long A, Albert S, Brennan M (1973). A human cell line from a pleural effusion derived from a breast carcinoma. J Natl Cancer Inst.

[49] Nakagawa M (1992). Reduced intracellular drug accumulation in the absence of P-glycoprotein (mdr1) overexpression in mitoxantrone-resistant human MCF-7 breast cancer cells. Cancer Res.

[50] Berridge MV, Tan AS (1993). Characterization of the cellular reduction of 3-(4,5-dimethylthiazol-2-yl)-2,5-diphenyltetrazolium bromide (MTT): subcellular localization, substrate dependence, and involvement of mitochondrial electron transport in MTT reduction. Arch Biochem Biophys.

[51] Kupcsik L (2011). Estimation of cell number based on metabolic activity: the MTT reduction assay. Methods Mol Biol.

[52] Sharom FJ (2011). The P-glycoprotein multidrug transporter. Essays in biochemistry.

[53] Ambudkar SV (1999). Biochemical, cellular, and pharmacological aspects of the multidrug transporter. Annual review of pharmacology and toxicology.

[54] Cripe LD (2010). Zosuquidar, a novel modulator of P-glycoprotein, does not improve the outcome of older patients with newly diagnosed acute myeloid leukemia: a randomized, placebo-controlled trial of the Eastern Cooperative Oncology Group 3999. Blood.

[55] Taylor NMI (2017). Structure of the human multidrug transporter ABCG2. Nature.

[56] Johnson ZL, Chen J (2017). Structural Basis of Substrate Recognition by the Multidrug Resistance Protein MRP1. Cell.

[57] Hamilton TC, Young RC, Ozols RF (1984). Experimental model systems of ovarian cancer: applications to the design and evaluation of new treatment approaches. Seminars in oncology.

[58] Shum D (2008). A high density assay format for the detection of novel cytotoxic agents in large chemical libraries. J Enzyme Inhib Med Chem.

[59] Berridge MV, Tan AS, McCoy KD, Wang R (1996). The biochemical and cellular basis of cell proliferation assays that use tetrazolium salts. Biochemica.

[60] Huyck L, Ampe C, Van Troys M (2012). The XTT cell proliferation assay applied to cell layers embedded in three-dimensional matrix. Assay Drug Dev Technol.

[61] Suda K (2017). Primary Double-Strike Therapy for Cancers to Overcome EGFR Kinase Inhibitor Resistance: Proposal from the Bench. Journal of thoracic oncology: official publication of the International Association for the Study of Lung Cancer.

[62] Tangutur, A. D. *et al*. Microtubule Targeting Agents as Cancer Chemotherapeutics: An Overview of Molecular Hybrids as Stabilising and Destabilising Agents. *Curr Top Med Chem* (2017). 10.2174/1568026617666170104145640 28056738

[63] Weaver BA (2014). How Taxol/paclitaxel kills cancer cells. Molecular biology of the cell.

[64] Woods CM, Zhu J, McQueney PA, Bollag D, Lazarides E (1995). Taxol-induced mitotic block triggers rapid onset of a p53-independent apoptotic pathway. Mol Med.

[65] Elmore S (2007). Apoptosis: a review of programmed cell death. Toxicol Pathol.

[66] Jordan MA (2002). Mechanism of action of antitumor drugs that interact with microtubules and tubulin. Curr Med Chem Anticancer Agents.

[67] Feller N, Broxterman HJ, Wahrer DC, Pinedo HM (1995). ATP-dependent efflux of calcein by the multidrug resistance protein (MRP): no inhibition by intracellular glutathione depletion. FEBS Lett.

[68] Legrand O, Simonin G, Perrot JY, Zittoun R, Marie JP (1998). Pgp and MRP activities using calcein-AM are prognostic factors in adult acute myeloid leukemia patients. Blood.

[69] Legrand O, Simonin G, Perrot JY, Zittoun R, Marie JP (1999). Both Pgp and MRP1 activities using calcein-AM contribute to drug resistance in AML. Adv Exp Med Biol.

[70] Pusztai L (2005). Phase II study of tariquidar, a selective P-glycoprotein inhibitor, in patients with chemotherapy-resistant, advanced breast carcinoma. Cancer.

[71] Kelly RJ (2011). A pharmacodynamic study of docetaxel in combination with the P-glycoprotein antagonist tariquidar (XR9576) in patients with lung, ovarian, and cervical cancer. Clin Cancer Res.

[72] Boylan KL (2011). Claudin 4 is differentially expressed between ovarian cancer subtypes and plays a role in spheroid formation. International journal of molecular sciences.

[73] Stordal B (2012). Resistance to paclitaxel in a cisplatin-resistant ovarian cancer cell line is mediated by P-glycoprotein. PLoS One.

[74] Demeule M, Brossard M, Beliveau R (1999). Cisplatin induces renal expression of P-glycoprotein and canalicular multispecific organic anion transporter. Am J Physiol.

[75] Di Pietro A (2002). Modulation by flavonoids of cell multidrug resistance mediated by P-glycoprotein and related ABC transporters. Cell Mol Life Sci.

[76] Badhan R, Penny J (2006). In silico modelling of the interaction of flavonoids with human P-glycoprotein nucleotide-binding domain. Eur J Med Chem.

[77] Syed SB (2017). Targeting P-glycoprotein: Investigation of piperine analogs for overcoming drug resistance in cancer. Scientific reports.

[78] Stone KR, Mickey DD, Wunderli H, Mickey GH, Paulson DF (1978). Isolation of a human prostate carcinoma cell line (DU 145). Int J Cancer.

[79] Laemmli UK (1970). Cleavage of structural proteins during the assembly of the head of bacteriophage T4. Nature.

[80] Rampersad SN (2012). Multiple applications of Alamar Blue as an indicator of metabolic function and cellular health in cell viability bioassays. Sensors (Basel).

[81] Ma MT (2013). MiR-487a resensitizes mitoxantrone (MX)-resistant breast cancer cells (MCF-7/MX) to MX by targeting breast cancer resistance protein (BCRP/ABCG2). Cancer Lett.

[82] Rasband, W. S. (U. S. National Institutes of Health, Bethesda, Maryland, USA; 1997–2017).

[83] Friedrich J, Seidel C, Ebner R, Kunz-Schughart LA (2009). Spheroid-based drug screen: considerations and practical approach. Nat Protoc.

[84] Wu C-Y (2012). Studies toward the unique pederin family member psymberin: structure–activity relationships, biochemical studies, and genetics identify the mode-of-action of psymberin. Journal of the American Chemical Society.

