# Supplementary material for: Targeted inhibitors of P-glycoprotein increase chemotherapeutic-induced mortality of multidrug resistant tumor cells
Source: Sci Rep. 2018 Jan 17;8:967. doi: 10.1038/s41598-018-19325-x (PMC5772368; doi:10.1038/s41598-018-19325-x)
Supplement: Supplementary file 1 — Supplementary Information [file 41598_2018_19325_MOESM1_ESM.pdf]

Supplemental information

for

Targeted inhibitors of P-glycoprotein increase chemotherapeutic-induced mortality of multidrug resistant tumor cells

AUTHORS: Amila K. Nanayakkara[1], Courtney A. Follit[1], Gang Chen[1], Noelle S. Williams[2], Pia D. Vogel[1]*, John G. Wise[1]*

[1]From the Center for Drug Discovery, Design and Delivery, the Center for Scientific Computing, and the Department of Biological Sciences, Southern Methodist University, Dallas, TX, USA 75275-0376 U.S.A.;

[2]Department of Biochemistry, UT Southwestern Medical Center, 5323 Harry Hines Boulevard, Dallas, Texas 75390-9038, United States

* Corresponding authors: pvogel@smu.edu, 214-768-1790 (phone) or jwise@smu.edu, 214-768-3426 (phone); 214-768-3955 (fax)

Figure S1:

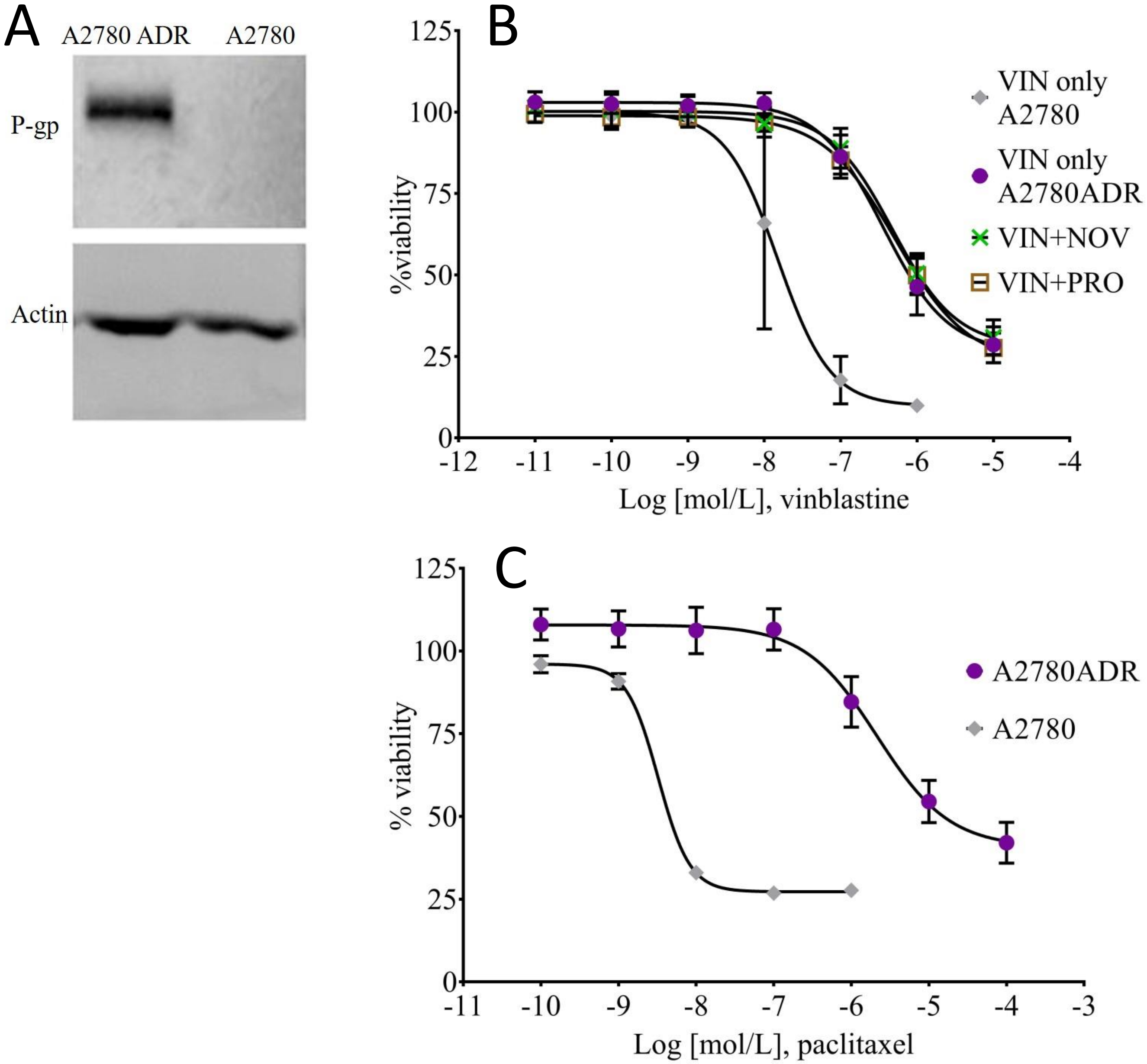

Figure S1 – The multidrug resistance phenotype of an ovarian cancer cell line. Panel A: Western blot analyses of A2780ADR (left lane) and A2780 (right lane) were performed using anti-P-gp and anti-actin antibodies. **Panel B:** Cell viability was determined using the resazurin assay to analyze the resistance to vinblastine; *diamonds*, chemotherapeutic-sensitive cell line A2780; *circles*, chemotherapy-resistant cell line A2780ADR; *open squares*, addition of 250µM of the MRP1-specific inhibitor, probenecid; *crosses*, addition of 60µM of the BCRP-specific inhibitor, novobiocin. **Panel C**: Cell viability assays as in Panel B, but in the presence of paclitaxel; *diamonds*, chemo-sensitive cell line A2780; *circles*, chemotherapy-resistance cell line A2780ADR. Data represents the mean ± SD of 12 replicates from two independent experiments. Some error bars are too small to be visible outside of the data points. PTX, paclitaxel; VIN, vinblastine; NOV, novobiocin; PRO, probenecid.

Figure S2:

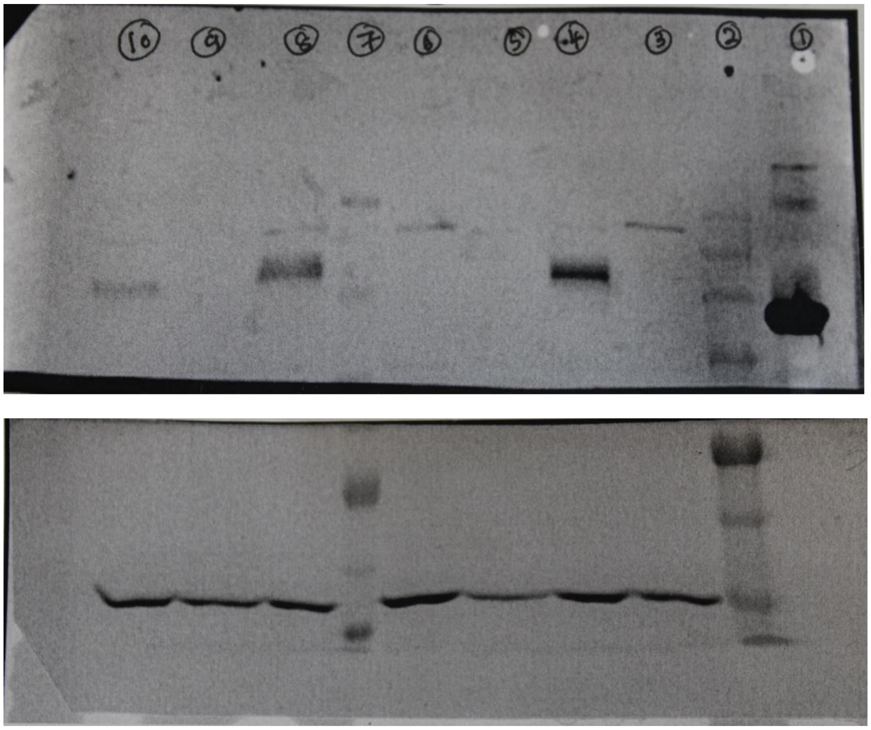

Figure S2 – Data for Figure S1: Original Western blot analysis of protein expression in cell lysates from prostate cancer cell lines A2780 (drug-sensitive) and A2780 ADR (multidrug resistant)

A single SDS gel was run as described in Methods and then cut in two parts for probing with two different primary antibodies. **Top panel:** Western blot analysis of A2780ADR and A2780 was performed using anti P-gp antibody C219 (from Enzo Life Sciences, NY). **Bottom panel:** The lower half of the same gel was probed with β-actin monoclonal antibody C4 (from Santa Cruz Biotechnology, CA).

The proteins loaded were of course identical in both top and bottom panels:

Lanes 1 through 10 were marked numerically with a black pen.

Lane 1 – Control, purified P-gp protein isolated from *Pichia pastoris*, 0.5 µg

Lane 2 - New England Bio Lab color pre stained plus protein ladder (catalog # P7712), 50 µg; Visible bands from top to bottom in the two panels represent 245, 190, 135, 100, 80, 58, 46, 32 kDa, respectively.

Lane 3 – total cell extract from cell line A2780 (drug sensitive prostate cancer cell line), 50 µg

Lane 4 – total cell extract from cell line A2780 ADR (multidrug resistant prostate cancer cell line), 50 µg

Lanes 5 to 10 were samples that were not relevant to this study.

We note that the difference in mobility between the *Pichia*-derived murine MDR3 P-gp (lane 1) and that of the human cancer cell-derived Pgp (lane 4) likely resides in differences in both sequence and glycosylation states.

Figure S3:

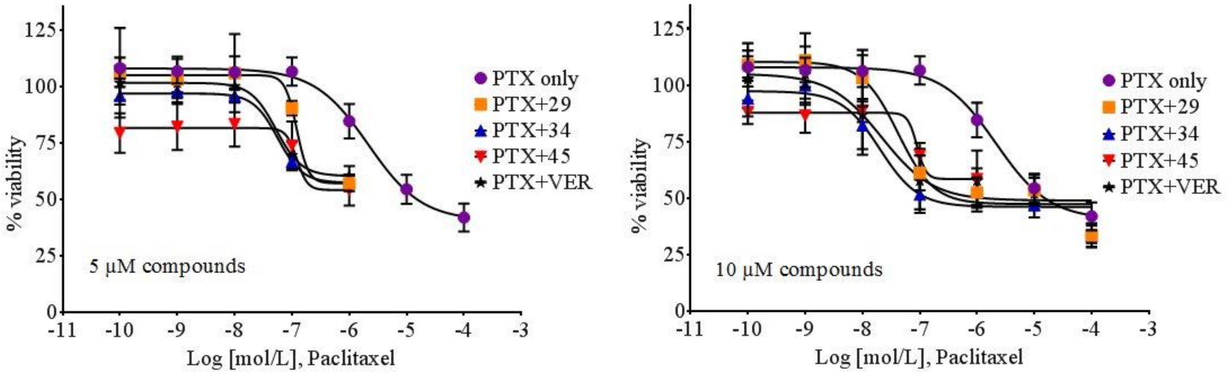

Figure S3 – Reversal of paclitaxel resistances by novel inhibitors of P-glycoprotein using metabolic viability assays. The A2780ADR cells were treated in the presence of compounds 29, 34, 45 or verapamil with the indicated concentrations of paclitaxel. **Panel A:** 5 µM of experimental P-gp inhibitor compounds were used. **Panel B:** 10 µM of compounds were used. Compare with Figure 1 of the paper where 25 µM experimental compounds were used. *Circles*, paclitaxel alone; *squares*, paclitaxel plus compound 29; *triangles*, paclitaxel compound 34; *inverted triangles*, paclitaxel plus compound 45; *stars*, paclitaxel plus P-gp substrate verapamil. Data represents the mean ± SD of 12 replicates from two independent experiments. PTX, paclitaxel; VER, verapamil.

Figure S4

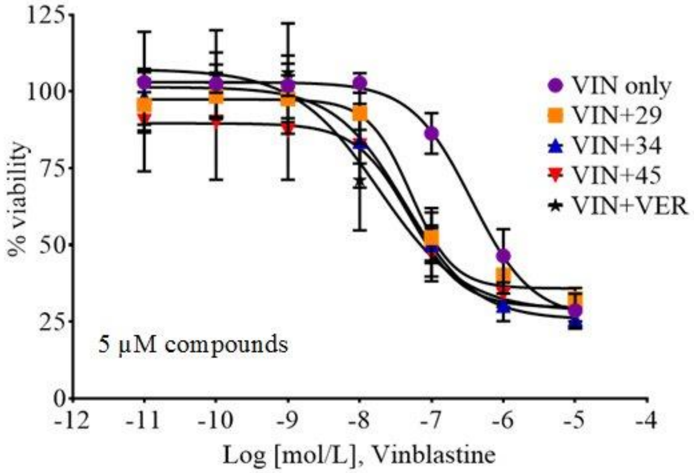

Figure S4 – Reversal of vinblastine resistances by novel inhibitors of P-glycoprotein using metabolic viability assays. The A2780ADR cells were treated in the presence of compounds 29, 34, 45 or verapamil with the indicated concentrations of vinblastine. 5 µM of experimental P-gp inhibitor compounds were used. *circles*, vinblastine alone; *squares*, vinblastine plus compound 29; *triangles*, vinblastine plus compound 34; *inverted triangles*, vinblastine plus compound 45; *stars*, vinblastine plus P-gp substrate verapamil. Data represents the mean ± SD of 12 replicates from two independent experiments. VIN, vinblastine; VER, verapamil.

Figure S5:

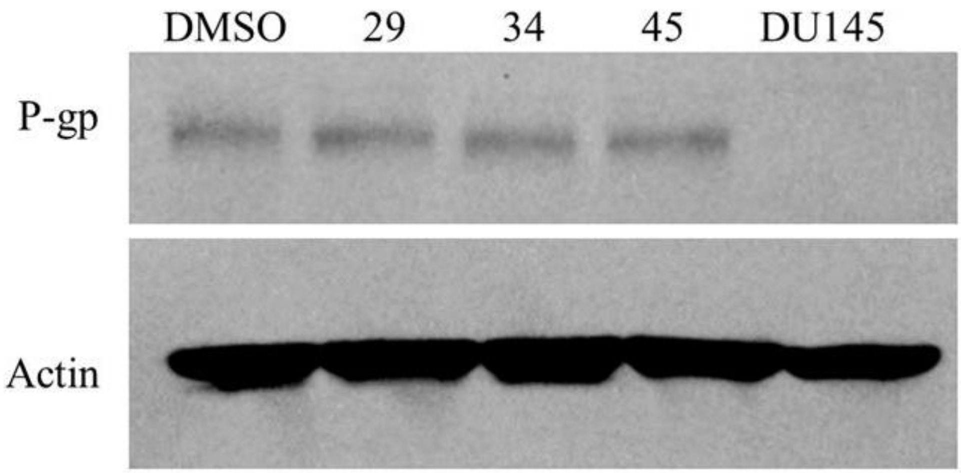

Figure S5 - P-gp expression is unchanged by compound treatment in DU145 TXR prostate cancer cell line. Western blots of DU145 TXR cancer cells treated with 5 μM of compounds (29, 34 or 45) and vehicle/DMSO were performed using anti-P-gp and anti-actin antibodies. 50 µg of cell lysates were loaded in each lane. Analyses were performed using anti-P-gp antibody C219 from Enzo Life Sciences, NY (top) and anti-β-actin antibody from Santa Cruz Biotechnology, CA (bottom) as described in Methods. P-gp was not detected in the parental drug sensitive DU145 prostate cancer cell line.

Figure S6:

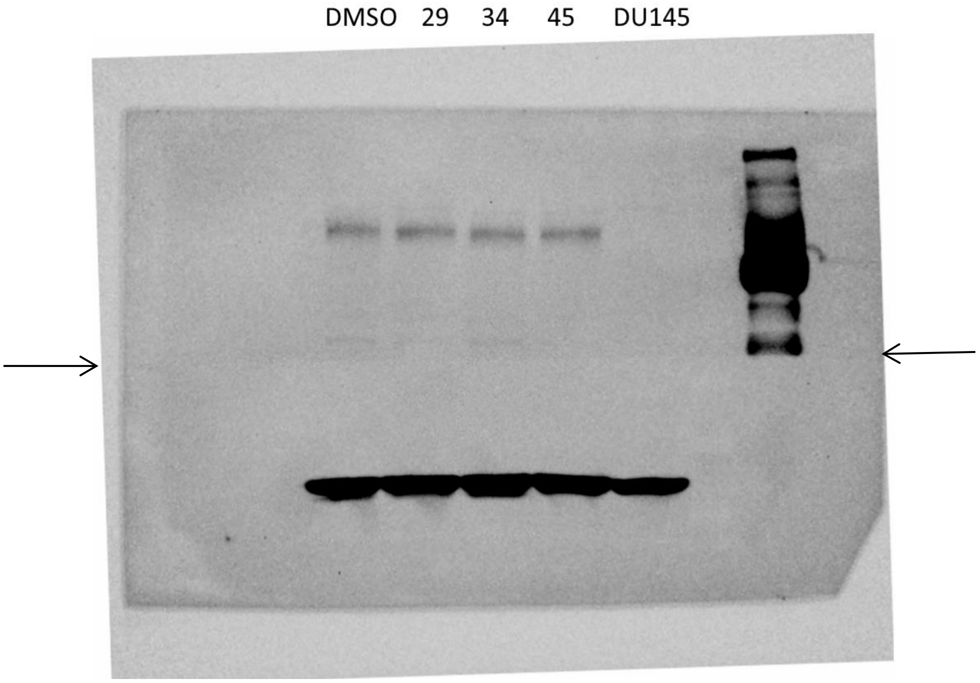

Figure S6 – Original Western blot photograph for Figure S5. P-gp expression was unchanged by compound treatment in DU145 TXR prostate cancer cell line. Western blots of DU145 TXR cancer cell lysates (50 µg/lane) from cultures treated with vehicle/DMSO or 5 μM of compounds (29, 34 or 45) as indicated. Analyses were performed using anti-P-gp antibody C219 from Enzo Life Sciences, NY (top) and anti-β-actin antibody from Santa Cruz Biotechnology, CA (bottom) as described in Methods. P-glycoprotein was not detected in the parental drug sensitive prostate cancer cell line ("DU145" lane). Right most lane was 10 μg of semi-purified P-gp isolated from a *Pichia pastoris* strain expressing mouse P-gp. The two arrows indicate where the gel-blot was cut for probing with the two different antibodies. The two halves were put back together before recording the image.

Figure S7:

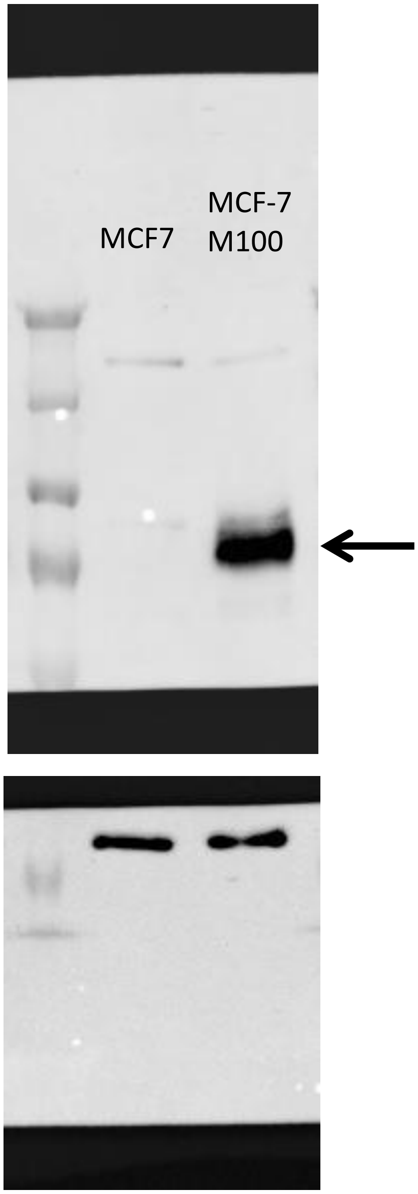

Figure S7 – Original Western blot analysis of protein expression in cell lysates from breast cancer cell lines MCF-7 (drug sensitive) and MCF-7 M100 (multidrug resistant). A single SDS gel was run as described in Methods and then cut in two parts for probing with two different primary antibodies.

Top panel: Western blot analysis of MCF-7 and MCF-7 M100 was performed using anti BCRP antibody B1 (from Santa Cruz Biotechnology, CA).

Bottom panel: The lower half of the same gel was probed with β-actin monoclonal antibody C4 (from Santa Cruz Biotechnology, CA). The proteins loaded were of course identical in both top and bottom panels:

Lane #1 - Spectra™ Multicolor Broad Range Protein Ladder (catalog # 26623), Visible bands from top to bottom in the two panels represent 260, 140, 100, 70, 50, 40, 35 kDa, respectively.

Lane #2 – total cell extract from cell line MCF-7 (drug sensitive breast cancer cell line), 5 µg

Lane #3 – total cell extract from cell line MCF-7 M100 (multidrug resistant breast cancer cell line), 5 µg
